# Marine-derived peptides from *Rapana venosa* inhibit breast cancer cell growth through synergistic mechanisms with doxorubicin

**DOI:** 10.1038/s41598-025-18052-4

**Published:** 2025-09-12

**Authors:** Mai A. Saleh, Hassan M. M. Masoud, Mohamed R. Habib, Sherif R. AbdElGhany, Maggie E. Amer, Mohamed Fathy Abouel-Nour

**Affiliations:** 1 Zoology Department, Faculty of Science, Mansoura University, Mansoura, Dakahlia Governorate Egypt; 2 Molecular Biology Department, National Research Centre, 33 El-Buhouth St., Dokki, Giza 12622 Egypt; 3 Proteome Research Laboratory, Central Laboratories Network and Centers of Excellence, National Research Centre, 33 El-Buhouth St., Dokki, Giza 12622 Egypt; 4 Medical Malacology Department, Theodor Bilharz Research Institute, 1 Corniche El Nile St., Warrak El-Haddar, Imbaba, Giza 12411 Egypt

**Keywords:** *Rapana venosa*, Marine peptides, Breast cancer, Apoptosis, Cell cycle, Doxorubicin, Biochemistry, Biotechnology, Cancer, Cell biology, Drug discovery, Oncology

## Abstract

Marine natural products are a promising source of novel anticancer agents. This study evaluated the antitumor activity of peptide fractions derived from the marine gastropod *Rapana venosa* against human breast cancer cell lines MCF-7 (estrogen receptor-positive) and MDA-MB-231 (triple-negative). Peptides were isolated by enzymatic hydrolysis and Fast Protein Liquid Chromatography. All seven isolated fractions were systematically screened against four human cancer cell lines (MCF-7, MDA-MB-231, CaCo-2, and HepG2) to assess cancer type selectivity. Cytotoxicity was assessed using MTT assay, while cell cycle progression and apoptosis were analyzed by flow cytometry. Gene expression changes were examined by RT-qPCR. Two peptide fractions, RV1 and RV2, demonstrated remarkable selectivity for breast cancer cells, exhibiting 25–95-fold higher potency compared to other cancer types. These fractions showed significant dose-dependent cytotoxicity with IC_50_ values of 6.887–7.288 μg/ml (RV1) and 4.886–6.268 μg/ml (RV2) against breast cancer cells. Both fractions induced G0/G1 cell cycle arrest and promoted apoptosis through multiple pathways, upregulating pro-apoptotic genes (*TP53*, *AIFM1*, *CASP3*, *BAX*) and downregulating anti-apoptotic markers (*BCL2*, *miR-155*). Most significantly, combination treatments with doxorubicin resulted in remarkable synergistic effects, with RV2 + doxorubicin achieving 78.9% total apoptosis compared to 33.5% with doxorubicin alone. These results indicate that *R. venosa*-derived peptides exert selective anticancer effects against breast cancer cells through multiple mechanisms. The observed selectivity and synergism with doxorubicin suggest their potential as targeted adjuvant agents in combination chemotherapy. Further structural characterization and in vivo studies are needed to advance their therapeutic development.

**Supplementary Information:**

The online version contains supplementary material available at 10.1038/s41598-025-18052-4.

## Introduction

Breast cancer is a major global health challenge, with high incidence and mortality rates. In 2020 alone, cancer caused nearly 10 million deaths globally, with breast cancer being the most frequently diagnosed malignancy in females (24.2% of new cases)^1–3^. Despite advances in treatment, breast cancer is a heterogeneous disease comprising multiple molecular subtypes with differing prognoses and therapeutic responses. The luminal subtypes (often estrogen receptor-positive) generally respond to hormonal therapies, whereas HER2-enriched tumors are treated with anti-HER2 targeted agents^4,5^. On the other hand, basal-like or triple-negative breast cancers (TNBC) do not express estrogen receptor (ER), progesterone receptor (PR), and HER2. Therefore, this subtype of breast cancer has no targeted therapies and with poorer prognosis. Moreover, it is aggressive and prone to drug resistance, making TNBC an urgent focus for novel therapeutic approaches^6,7^.

Standard breast cancer interventions including surgery, radiation, chemotherapy, and endocrine or targeted therapies, have improved patient outcomes but still face significant limitations^8–10^. Cytotoxic chemotherapy, for example, non-selectively targets rapidly proliferating cells and often causes severe toxicity to healthy tissues, leading to side effects and compromised patient immune function^11,12^. Moreover, cancers can evolve resistance to chemotherapy and targeted drugs, rendering treatments ineffective over time^13,14^. Thus, there is an urgent need for novel anticancer drugs that are more effective and selective, while minimizing off-target effects^15,16^.

In this context, natural products have long been a cornerstone of drug discovery. It is estimated that more than 60% of current anticancer drugs are derived from or inspired by natural sources^16–18^. Oncolytic peptides represent particularly promising candidates for anticancer treatments, with multiple oncolytic peptides having entered clinical trials for various cancers, including breast cancer. These peptides demonstrate novel mechanisms of action including membrane disruption, immune system activation, and selective tumor cell targeting^19–21^. Peptide-based therapies have shown promise as potential anticancer agents due to their diverse mechanisms of action and lower toxicity profiles^22–24^. Anticancer peptides, often derived from host defense peptides, can preferentially kill tumor cells while sparing normal tissue^25–27^.

In recent years, the marine environment has emerged as a promising frontier for drug discovery, including anticancer compounds^28^. Marine organisms often produce chemically novel metabolites and peptides for defense or communication. These bioactive compounds and peptides are a product of evolutionary adaptations to complex ecosystems, frequently exhibit unusual structures not found in terrestrial natural products^29–32^. Several marine-derived agents have reached clinical use as anticancer drugs^33,34^. Mollusks managed to establish themselves in hostile marine environments in the presence of viruses, bacteria, and predators because of their chemical defense and humoral elements including antioxidant enzymes and antimicrobial peptides^35–37^. These chemical components are found to be bioactive with anticancer or biomedical relevance^36,38^.

The mollusk *Rapana venosa*, a predatory sea snail, is a rich source of bioactive compounds with diverse biomedical applications, ranging from oncology to immunology and inflammation therapy^39–41^. The therapeutic value of *R. venosa*-derived compounds in drug development, as novel agents with higher specificity and lower toxicity shows promising anticancer potential. Previous studies have demonstrated that fractions from *R. venosa* hemolymph exhibit significant antitumor activity against various breast cancer cell lines and show strong synergistic effects when combined with chemotherapeutic drugs like cisplatin and tamoxifen^30^. However, the specific contribution of smaller peptides derived from enzymatic hydrolysis of *R. venosa* tissues to the overall anticancer potential remains largely unexplored.

This study was designed to investigate the antitumor potential of peptide fractions isolated from enzymatic hydrolysates of *R. venosa* tissue against human breast cancer cell lines. We specifically evaluated their effects against MCF-7 and MDA-MB-231 breast cancer cells. Our objectives included assessing the cytotoxic effects, elucidating mechanisms of action through cell cycle and apoptosis analysis, and examining the modulation of key regulatory genes involved in apoptosis, autophagy, and oncogenic pathways. Additionally, we investigated the potential synergistic effects of these peptides when combined with doxorubicin, a conventional chemotherapeutic agent, to explore their utility as adjuvant therapeutic agents in combination therapy protocols.

## Methods

### Sample collection and identification

Specimens of the marine snail *R. venosa* were collected manually from the intertidal and shallow subtidal zones (1–3 m depth) of the Mediterranean Sea. After collection, snails were washed with ambient seawater to remove sand and debris. Live specimens were transported to the Medical Malacology Department at Theodor Bilharz Research Institute (Giza, Egypt) in seawater-filled aquaria. For taxonomic identification, representative specimens were rinsed with sterile water, fixed in 2% buffered formaldehyde, and then transferred to absolute ethanol for preservation, following established protocols^42^. Species-level identification was confirmed using standard taxonomic keys^43^ and verified against the World Register of Marine Species (WoRMS) database^44^.

### Chemicals and reagents

Pepsin (porcine gastric mucosa), trypsin (porcine pancreas), and α-chymotrypsin (bovine pancreas) were purchased from Sigma-Aldrich (St. Louis, MO, USA). Dimethyl sulfoxide (DMSO), crystal violet stain, phenazine methosulfate (PMS), and trypan blue dye were also obtained from Sigma-Aldrich. Cell imaging reagents (Vibrant® CellTrace™ CFSE Cell Proliferation Kit, Cat. No. V13154) were sourced from Thermo Fisher Scientific and stored at 2–8 °C. Cell culture reagents, including Fetal Bovine Serum (FBS), RPMI-1640 medium, Dulbecco’s Modified Eagle Medium (DMEM), HEPES buffer solution, L-glutamine, gentamycin sulfate solution, and 0.25% Trypsin–EDTA solution, were purchased from Lonza AG (Basel, Switzerland). All other chemicals and solvents were of analytical grade.

### Peptide extraction and enzymatic hydrolysis

All extraction and hydrolysis procedures were conducted at 4 °C unless stated otherwise. Fresh *R. venosa* tissues (20 g wet weight) were combined with 1.5 volumes (30 ml) of 0.1 M potassium phosphate buffer (pH 6.4). The mixture was homogenized using a Teflon-pestle homogenizer. The homogenate was centrifuged at 10,000 ×*g* for 20 min at 4 °C, and the supernatant (crude protein extract) was collected. A sequential enzymatic hydrolysis protocol, adapted from Nazeer et al.^45^, was employed. The pH of the crude extract was adjusted to 2.5 using HCl. Pepsin was added at an enzyme-to-substrate (E:S) ratio of 1:100 (w/w), and the mixture was incubated with shaking at 37 °C for 2 h. Subsequently, the pH was adjusted to 8.0 using NaOH. Both trypsin and α-chymotrypsin were added, each at an E:S ratio of 1:100 (w/w), and the incubation continued with shaking at 37 °C for an additional 2.5 h.

To stop the enzymatic reactions, the pH of the hydrolysate was adjusted to 6.5, and the sample was immediately heated in a boiling water bath (100 °C) for 10 min to denature and inactivate the proteases. The heat-treated sample was then centrifuged at 10,000 ×*g* for 15 min at 4 °C to remove any precipitated material. The final supernatant, containing the peptide hydrolysate, was collected, rapidly frozen at − 80 °C, and subsequently lyophilized to obtain a dry powder for storage and further analysis.

### Fast protein liquid chromatography (FPLC)

The hydrolyzed *R. venosa* peptide sample was fractionated using an ÄKTA Avant 150 FPLC system (GE Healthcare Life Sciences) at the Central Laboratories Network, National Research Centre, Cairo, Egypt. Before chromatography, the lyophilized hydrolysate was reconstituted in binding buffer A (20 mM sodium acetate, pH 4.0) at a concentration of 10 mg/ml and filtered through a 0.22 μm membrane filter. Fractionation was performed using a HiTrap DEAE FF anion exchange column (1 ml bed volume, GE Healthcare) equilibrated with 5 column volumes of binding buffer A. The sample (1 ml) was loaded onto the column at a flow rate of 0.5 ml/min. Unbound material was washed with 5 column volumes of binding buffer A. Bound peptides were eluted using a linear gradient of 0.0–1.5 M NaCl in binding buffer A over 20 column volumes at a flow rate of 1 ml/min. The process was monitored by measuring absorbance at 280 nm. Individual fractions (1 ml each) were collected, and those corresponding to distinct chromatographic peaks were pooled, desalted using PD-10 desalting columns (GE Healthcare), lyophilized, and stored at − 20 °C. The major bioactive fractions were designated as 1A, 1B, 1C, 2A, 3A1 (RV1), 3A5 (RV2), and 3B based on their elution order.

### Electrophoretic analysis

Sodium dodecyl sulfate–polyacrylamide gel electrophoresis (SDS-PAGE) was performed according to Laemmli^46^ to analyze the protein profile of the crude extract and the enzymatic hydrolysate. Samples were mixed with 4X Laemmli sample buffer and heated at 95 °C for 5 min. Electrophoresis was conducted using a vertical mini-gel system (Bio-Rad, USA) with 12% resolving gel and 5% stacking gel at a constant voltage of 120 V. A molecular weight marker (Precision Plus Protein™ Dual Color Standards, Bio-Rad) was included. Gels were stained with Coomassie Brilliant Blue R-250 for 1 h and destained until protein bands were clearly visible. Gel images were captured using a gel documentation system (Bio-Rad) and analyzed using Image Lab™ software (Bio-Rad).

## Identification and characterization of peptides

### Scanning electron microscopy (SEM)

The morphological characteristics of lyophilized peptide fractions RV1 and RV2 were examined using an ISI-60 scanning electron microscope operated at 25–36 kV in backscatter electron mode. Samples were prepared by mounting a small amount of each peptide fraction on aluminum stubs using double-sided carbon tape. The mounted samples were sputter-coated with a thin layer of gold (approximately 15 nm thickness) using a sputter coater (JEOL JFC-1100E). Multiple fields were examined, and representative micrographs were captured at various magnifications. Particle size measurements were performed directly on the micrographs using the microscope’s calibrated measurement software. The SEM analysis was conducted at the Electron Microscope Unit, Faculty of Science, Mansoura University, Mansoura, Egypt.

### Proton nuclear magnetic resonance (^1^H NMR) spectroscopy

^1^H NMR spectra of peptide fractions RV1 and RV2 were recorded using a Jeol ECA-500II spectrophotometer operating at 500 MHz. Samples were prepared by dissolving approximately 5 mg of each lyophilized peptide fraction in 0.6 ml of deuterium oxide (D_2_O, 99.9% D). Tetramethylsilane (TMS) was used as an internal reference standard. The spectra were acquired at 25 °C with a spectral width of 10 ppm, 128 scans, acquisition time of 3.0 s, and a relaxation delay of 1.0 s. The resulting spectra were processed using Delta NMR software (version 5.0.4, JEOL). Chemical shifts (δ) were reported in parts per million (ppm) relative to TMS. The NMR analysis was performed at the Chemistry Department, Faculty of Science, Mansoura University, Mansoura, Egypt.

### Amino acid analysis

The amino acid composition of peptide fractions RV1 and RV2 was determined following acid hydrolysis and high-performance liquid chromatography (HPLC) analysis. For each fraction, 0.1 g of lyophilized sample was hydrolyzed with 5 ml of 6 M HCl in sealed, evacuated glass tubes at 120 °C for 24 h. After hydrolysis, samples were filtered through 0.22 μm membrane filters. A 1 ml aliquot of each filtrate was dried under vacuum and reconstituted in 1 ml of 0.1 M HCl for HPLC analysis. Amino acid analysis was performed using an Agilent 1260 series HPLC system (Agilent Technologies, USA) at the Central Laboratories Network, National Research Centre, Cairo, Egypt. Separation was achieved on an Eclipse Plus C8 column (4.0 mm × 250 mm i.d., 5 μm particle size) maintained at 40 °C. The mobile phase consisted of buffer A (40 mM sodium phosphate monobasic, pH 7.8) and buffer B (acetonitrile:methanol:water, 45:45:10, v/v/v). A gradient elution program was employed at a flow rate of 1 ml/min, as described by Bartolomeo and Maisano^47^ and Jajić et al.^48^. Detection was performed using a fluorescence detector with excitation at 340 nm and emission at 450 nm following pre-column derivatization with o-phthalaldehyde (OPA). Amino acid standards (Sigma-Aldrich) were used for identification and quantification. The amino acid content was expressed as a percentage of the total amino acids detected.

### Cell lines and culture conditions

The primary targets for this study were human breast adenocarcinoma cell lines MCF-7 and MDA-MB-231, obtained from the Medical Experimental Research Center (MERC), Faculty of Medicine, Mansoura University, Mansoura, Egypt. To assess the selectivity of isolated peptide fractions for breast cancer cells, additional human cancer cell lines were included as comparative controls: colorectal adenocarcinoma cell line CaCo-2 and hepatocellular carcinoma cell line HepG2, also obtained from MERC. MCF-7, MDA-MB-231, and CaCo-2 cells were routinely cultured in Dulbecco’s Modified Eagle Medium (DMEM; Lonza, Basel, Switzerland), while HepG2 cells were maintained in RPMI-1640 medium (Lonza, Basel, Switzerland). All media were supplemented with 10% (v/v) heat-inactivated Fetal Bovine Serum (FBS; Sigma-Aldrich, Germany), 100 U/ml penicillin, 100 μg/ml streptomycin, and 0.25 μg/ml amphotericin B (Antibiotic–Antimycotic solution; Lonza). All cell lines were maintained as monolayers in T-75 flasks in a humidified incubator at 37 °C with a 5% CO_2_ atmosphere and were subcultured every 2–3 days using 0.25% Trypsin–EDTA solution (Lonza) when they reached 80–90% confluency. Cell viability was routinely checked using the trypan blue exclusion assay, and cultures were regularly tested for mycoplasma contamination.

### Cell viability assay (MTT assay)

To identify the most potent bioactive fractions and establish their selectivity for breast cancer cells, all isolated peptide fractions (1A, 1B, 1C, 2A, 3B, RV1, RV2) were initially screened against four human cancer cell lines: MCF-7 and MDA-MB-231 (primary breast cancer targets), and CaCo-2 and HepG2 (selectivity controls representing different cancer types). The cytotoxic effects were assessed using the 3-(4,5-dimethylthiazol-2-yl)-2,5-diphenyltetrazolium bromide (MTT) assay. Based on the screening results demonstrating superior cytotoxic activity and preferential selectivity for breast cancer cells, fractions RV1 and RV2 were selected for comprehensive mechanistic studies, which were conducted exclusively on MCF-7 and MDA-MB-231 cells.

Cells were seeded into 96-well flat-bottom microplates at a density of 5 × 10^3^–7 × 10^3^ cells per well in 200 μl of complete culture medium and allowed to adhere for 24 h at 37 °C in a 5% CO_2_ incubator. Following attachment, the medium was replaced with fresh medium containing various concentrations of RV1, RV2, or DOXO. Control wells received medium only containing the vehicle. Cells were incubated with the treatments for 48 h and 72 h. After incubation, 10 μl of MTT solution (5 mg/ml in sterile PBS; Sigma-Aldrich) and 100 μl of media without phenol red were added to each well, and the plates were incubated for an additional 4 h at 37 °C. The medium containing MTT was then removed (85 μl), and the resulting formazan crystals were dissolved by adding 50 μl of Dimethyl Sulfoxide (DMSO; Sigma-Aldrich) to each well and incubating with gentle shaking for 15 min at room temperature. The absorbance was measured at 570 nm using a microplate reader. Background absorbance at 570 nm was subtracted where applicable. Cell viability was expressed as a percentage relative to the untreated control cells using the following formula:

$$\% \,{\text{Cell}}\,{\text{Viability}} = \left( {{\text{A}}_{570} \,{\text{of}}\,{\text{treated}}\,{\text{cells}}/{\text{A}}_{570} \,{\text{of}}\,{\text{control}}\,{\text{cells}}} \right) \times 100.$$

Experiments were performed in triplicate for each concentration and time point. The half-maximal inhibitory concentration (IC_50_), defined as the concentration required to inhibit cell viability by 50%, was calculated from the dose–response curves generated using non-linear regression analysis (e.g., using GraphPad Prism software, version 8.1). The calculated IC_50_ values were used to determine the concentrations for subsequent mechanistic studies.

### Cell cycle and apoptosis analysis

MCF-7 and MDA-MB-231 cells were seeded in tissue culture flask 25 cm^2^ at a density of 1 × 10⁶ cells and allowed to adhere overnight. Cells were then treated with RV1, RV2, or DOXO at their respective IC_50_ concentrations for 72 h. Control cells were treated with the vehicle only. Following treatment, both adherent and floating cells were collected by trypsinization, washed twice with ice-cold Phosphate-Buffered Saline (PBS), and fixed by dropwise addition into ice-cold 70% (v/v) ethanol while vortexing gently. Fixed cells were stored at − 20 °C for at least 2 h (or overnight). Prior to analysis, fixed cells were washed with PBS and resuspended in 500 μL of staining solution containing 50 μg/ml Propidium Iodide (PI; Sigma-Aldrich) and 100 μg/ml RNase A (Sigma-Aldrich) in PBS. Cells were incubated in the dark at room temperature for 30 min. Cell cycle distribution was analyzed using a flow cytometer (BD FACSAria™ III Flow Cytometer, CA-95131). The percentages of cells in the SubG1, G0/G1, S, and G2/M phases were quantified using cell cycle analysis software.

Apoptosis induction was assessed using Annexin V-FITC/PI Staining. Cells were categorized into four populations based on Annexin V-FITC and PI fluorescence: viable cells (Annexin V⁻/PI⁻), early apoptotic cells (Annexin V⁺/PI⁻), late apoptotic/necrotic cells (Annexin V⁺/PI⁺), and primarily necrotic cells (Annexin V⁻/PI⁺). Data analysis was performed using flow cytometry software (Flowjo v.9).

### Real-time quantitative PCR (RT-qPCR)

MCF-7 and MDA-MB-231 cells were seeded in tissue culture flask 25 cm^2^ and treated with RV1, RV2, or DOXO at their respective IC_50_ concentrations for 72 h. Total RNA was extracted from treated and control cells using the RNeasy® Mini Kit (Qiagen, Cat. No. 74104) according to the manufacturer’s protocol. RNA concentration and purity were assessed using a NanoDrop spectrophotometer (Thermo Fisher Scientific).

First-strand complementary DNA (cDNA) was synthesized from 1 μg of total RNA using the High-Capacity cDNA Reverse Transcription Kit (Applied Biosystems™, Thermo Fisher Scientific, Cat. No. 4368814) following the manufacturer’s instructions. The resulting cDNA was stored at − 20 °C until use. RT-qPCR was performed using a real-time PCR system (RT-PCR thermocyler, Cielo 6; CFX96, AIQ060) with SYBR® Green PCR Master Mix (Thermo Fisher Scientific, Lithuania). Each 20 μl reaction mixture contained 10 μl of 2 × SYBR Green Master Mix, 1 μL (10 pmol/μl final concentration) of each forward and reverse primer for the target gene or the housekeeping gene, 2 μl of cDNA template (diluted appropriately), and nuclease-free water to make up the final volume. Primer sequences for different studied genes are presented in (Table 1).

**Table 1:** Primer sequences, amplicon sizes, and annealing temperatures for all genes analyzed by quantitative real-time PCR (qRT-PCR). GAPDH was used as the housekeeping gene for normalization.

Gene	Forward primer	Reverse primer	Product size (bp)	Annealing temp (°C)
*miR-155*	TTAATGCTAATCGTGATAGGGGT	ATATGTAGGAGTCAGTTGGAGGC	55	61
*TP53*	CATAGTGTGGTGGTGCCCTATGAG	CAAAGCTGTTCCGTCCCAGTAGA	172	63
*AIFM1*	CAGAAAAAGGCCGCGTTATCT	ATACAATCAGTACCCTGGCCCC	160	59
*CASP3*	TTCCCAGGTTTTGTTTCCTG	CCTTTCACCGAAACAGCATT	143	61
*BCL2*	CTGCACCTGACGCCCTTCACC	CACATGACCCCACCGAACTCAAAGA	119	61
*BAX*	CGGGTTGTCGCCCTTTTCTA	TGGTTCTGATCAGTTCCGGC	82	60
*mTOR*	GCCCAGGCCGCATTGTCTCTAT	GCAGTAAATGCAGGTAGTCATCCAGGTT	84	62
*LC3A*	GCCTTCTTCCTGCTGGTGAAC	AGCCGTCCTCGTCTTTCTCC	91	58
*GAPDH* (housekeeping)	TGCACCACCAACTGCTTAGC	GGCATGGACTGTGGTCATGAG	87	59

The thermal cycling conditions were typically: initial denaturation at 95 °C for 10 min, followed by 40 cycles of denaturation at 95 °C for 15 s and annealing/extension at 60 °C for 1 min. A melt curve analysis was performed at the end of each run to confirm the specificity of the amplification product. All reactions were performed in triplicate. The relative expression levels of the target genes were normalized to the expression of the housekeeping gene GAPDH using the comparative cycle threshold (ΔΔCt) method^49^. The results were expressed as fold change relative to the untreated control group.

### Statistical analysis

Data from MTT assays, flow cytometry, and RT-qPCR experiments were analyzed using GraphPad Prism software (version 8.1.1, GraphPad Software, San Diego, CA, USA). All data are presented as the mean ± standard error (SE) of at least three independent experiments. Statistical significance between groups was determined using one-way analysis of variance (ANOVA) followed by Tukey’s post-hoc test for multiple comparisons. A *p* value of < 0.05 was considered statistically significant.

## Results

### Digestion of *R. venosa* snails and fractionation on FPLC

**Fig. 1:**
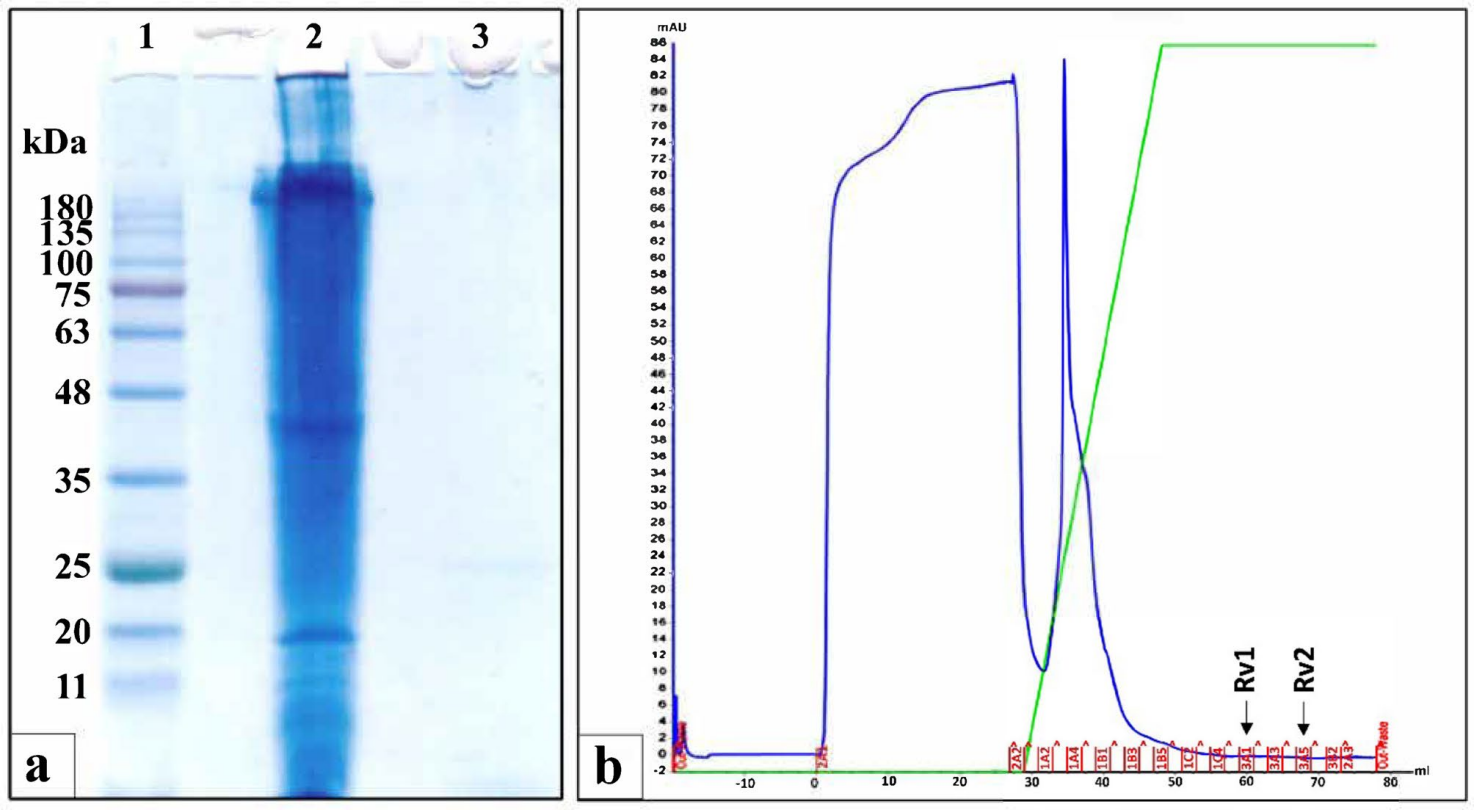
Enzymatic digestion of *Rapana venosa* tissue and FPLC fractionation of the hydrolysate: (**a**) SDS-PAGE analysis (12% gel, Coomassie G-250 staining) showing protein profiles. Lane 1: Molecular weight markers (kDa). Lane 2: Intact *R. venosa* crude extract before digestion. Lane 3: *R. venosa* hydrolysate after digestion with pepsin, trypsin, and α-chymotrypsin, demonstrating the effective hydrolysis of proteins into smaller peptides. (**b**) FPLC chromatogram (HiTrap DEAE FF anion exchange column) of the *R. venosa* hydrolysate. Peaks corresponding to collected fractions are indicated, including fractions 3A1 (RV1) and 3A5 (RV2) which were selected for further study based on their potent cytotoxic activity against breast cancer cell lines. The blue line represents absorbance at 280 nm, while the green line represents the gradient concentration of NaCl.

The *R. venosa* snail tissues were homogenized and subjected to sequential enzymatic hydrolysis using pepsin at pH 2.5, followed by trypsin and α-chymotrypsin at pH 8.0. SDS-PAGE analysis (Fig. 1a) of the intact *R. venosa* crude extract (lane 2) revealed numerous protein bands ranging from approximately 180 kDa down to 10 kDa. Following complete enzymatic digestion, these protein bands disappeared entirely, indicating effective hydrolysis into smaller peptides with no detectable protein bands remaining (lane 3). The resulting hydrolysate was then fractionated using FPLC on a HiTrap DEAE FF anion exchange column with a linear NaCl gradient (0.0–1.5 M). This chromatographic separation yielded seven distinct peptide fractions, designated 1A, 1B, 1C, 2A, 3B, 3A1 (RV1), and 3A5 (RV2), as indicated by the elution profile where RV1 and RV2 peaks are explicitly marked (Fig. 1b).

### Cytotoxic effects

To identify the most potent bioactive fractions and assess cancer type selectivity, all seven isolated peptide fractions (1A, 1B, 1C, 2A, 3B, RV1, RV2) were systematically screened for cytotoxic activity against four human cancer cell lines representing different cancer types: MCF-7 and MDA-MB-231 (breast cancer), CaCo-2 (colorectal carcinoma), and HepG2 (hepatocellular carcinoma) using the MTT assay (Table 2). The screening results revealed significant differences in both potency and cancer type selectivity among the fractions. Fractions RV1 and RV2 demonstrated the highest cytotoxic potency against breast cancer cell lines, with IC_50_ values ranging from 4.886 to 7.288 μg/ml. Specifically, RV1 showed IC_50_ values of 6.887 μg/ml (MCF-7) and 7.288 μg/ml (MDA-MB-231), while RV2 exhibited IC_50_ values of 6.268 μg/ml (MCF-7) and 4.886 μg/ml (MDA-MB-231). Importantly, both RV1 and RV2 demonstrated preferential selectivity for breast cancer cells compared to other cancer types. Against CaCo-2 cells, RV1 and RV2 showed significantly reduced activity with IC_50_ values of 429.012 and 211.032 μg/ml, respectively. Against HepG2 cells, the activity was further diminished, with IC_50_ values of 600.012 μg/ml (RV1) and 119.432 μg/ml (RV2). This represents 25–95-fold lower potency against non-breast cancer cell lines compared to breast cancer cells, indicating preferential selectivity for breast cancer.

**Table 2:** In vitro cytotoxicity IC_50_ (μg/ml) of *Rapana venosa* extracted peptide fractions against different human cancer cells.

Peptide fractions	MCF-7	MDA-MB-231	CaCO-2	HePG2
	IC_50_ (µg/ml)			
1A	501.4	275.6	892.1	348.5
1B	93.49	118.1	713.8	550.342
1C	699.98	500.41	253.763	501.876
3A1 (RV1)	6.887	7.288	429.012	600.012
2A	172.987	593.0	315.130	195.020
3B	98.86	283.1	202.007	232.807
3A5 (RV2)	6.268	4.886	211.032	119.432

In contrast, other peptide fractions showed minimal cytotoxic activity across all tested cell lines, with IC_50_ values generally exceeding 100 μg/ml. Fraction 1A showed moderate activity with IC_50_ values of 501.4 μg/ml (MCF-7) and 275.6 μg/ml (MDA-MB-231), while fractions 1B, 1C, 2A, and 3B exhibited IC_50_ values ranging from 93.49 to 699.98 μg/ml across different cell lines, with no consistent pattern of selectivity (Table 2). Based on these comprehensive screening results, fractions RV1 and RV2 were selected for detailed mechanistic studies due to their superior cytotoxic potency, preferential selectivity against breast cancer cell lines, and potential therapeutic relevance.

**Fig. 2:**
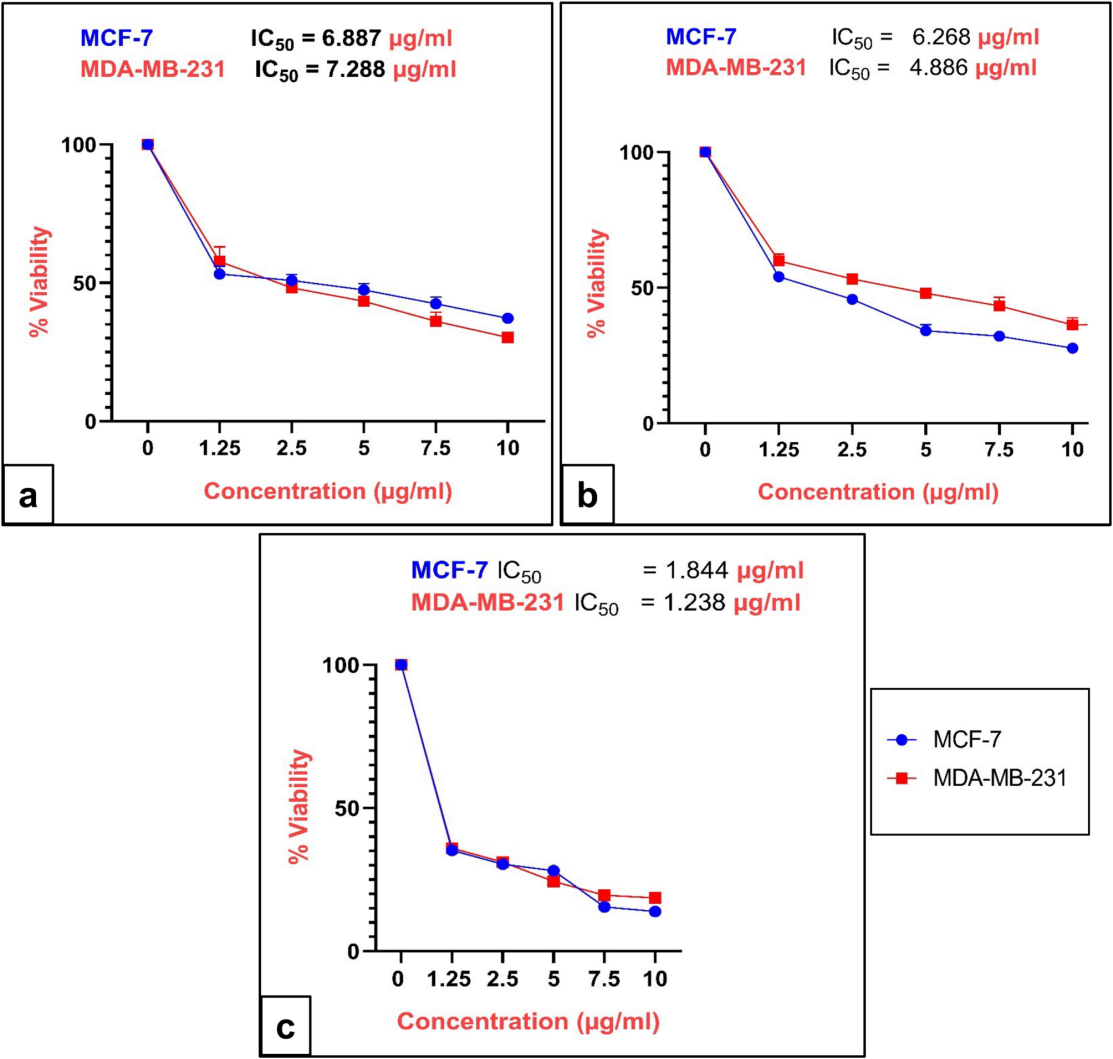
Cytotoxic effects of peptide fractions RV1, RV2, and doxorubicin (DOXO) on human breast cancer cell lines: Cell viability was assessed using the MTT assay after 72 h of treatment. Dose–response curves illustrate the percentage of cell viability relative to untreated controls for (**a**) RV1, (**b**) RV2, and (**c**) DOXO against MCF-7 and MDA-MB-231 cells. Data points represent the mean ± standard deviation (SD) of three independent experiments. IC_50_ values are detailed in the main text.

Following the identification of RV1 and RV2 as the most promising bioactive fractions, comprehensive dose–response analysis was performed specifically on breast cancer cell lines to determine precise IC_50_ values and assess time-dependent effects. The dose-dependent cytotoxicity of RV1, RV2, and the positive control drug doxorubicin (DOXO) was assessed after 48 and 72 h of treatment. Figure 2 presents the dose–response curves, demonstrating a clear dose-dependent decrease in cell viability for both peptides and the control drug across the tested concentration ranges (0.5–10 µg/ml).

Detailed analysis confirmed the potency of both fractions against breast cancer cells. For RV1, the calculated IC_50_ values were 6.887 µg/ml against MCF-7 cells and 7.288 µg/ml against MDA-MB-231 cells (Fig. 2a). RV2 demonstrated higher potency with IC_50_ values of 6.268 µg/ml against MCF-7 cells and 4.886 µg/ml against MDA-MB-231 cells (Fig. 2b). Both peptides showed moderate cytotoxic activity compared to the control drug DOXO (Fig. 2c), which exhibited IC_50_ values of 1.844 µg/ml and 1.238 µg/ml against MCF-7 and MDA-MB-231 cells, respectively. Notably, RV2 showed preferential activity against the triple-negative MDA-MB-231 cell line compared to the estrogen receptor-positive MCF-7 cells, suggesting potential efficacy against this challenging breast cancer subtype.

### Scanning electron microscopy (SEM)

**Fig. 3:**
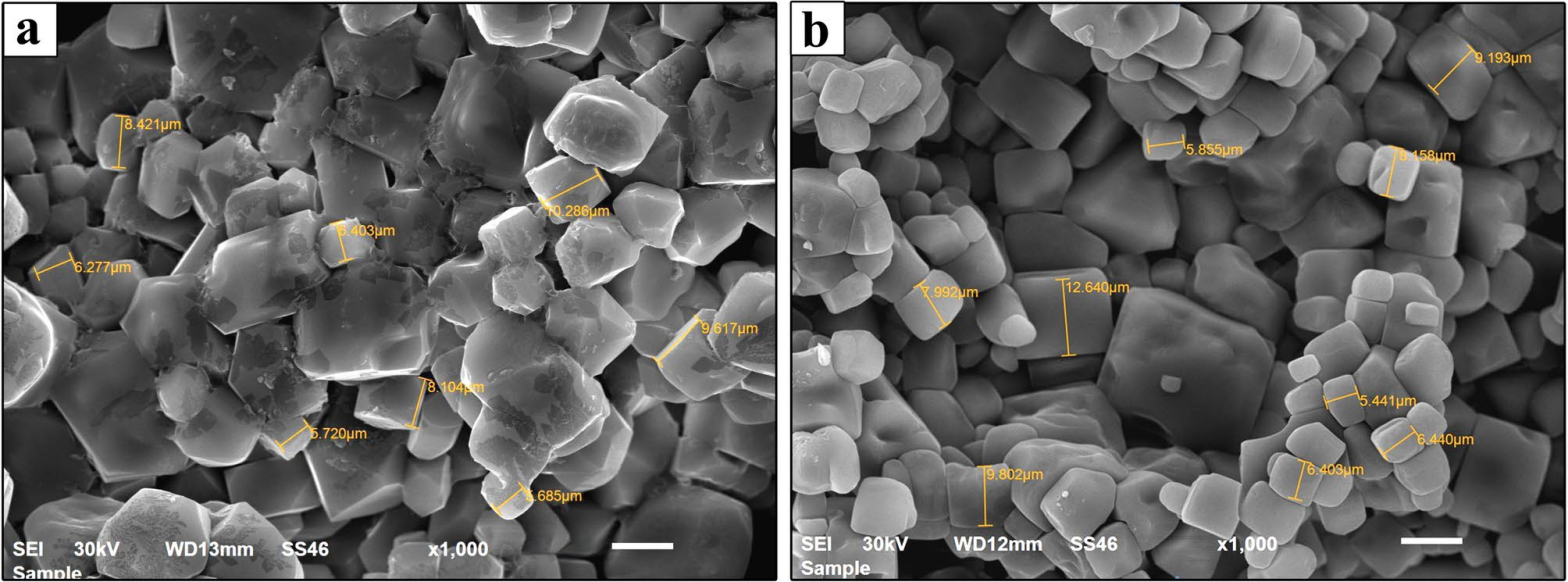
Scanning electron microscopy analysis of peptide fractions: (**a**) RV1 and (**b**) RV2. SEM images were obtained from lyophilized fractions prepared at 1 mg/mL concentration prior to drying and sputter coating and imaged at 1000× magnification with 30 kV acceleration voltage. Scale bars = 10 μm.

The morphological characteristics of peptides RV1 and RV2 were examined using SEM to investigate their physical properties and aggregation patterns (Fig. 3). The SEM analysis revealed distinct morphological differences between the two peptide fractions. RV1 particles appeared predominantly angular and block-like in shape, with well-defined edges and some clustering behavior. Particle size measurements indicated dimensions ranging from approximately 5.72 µm to 10.33 µm. The surfaces appeared relatively rough with distinct crystalline-like facets, and the particles showed a tendency to form aggregates (Fig. 3a).

In contrast, RV2 particles displayed more heterogeneous morphologies, including both faceted and rounded surfaces with smoother textures. The particle sizes ranged from approximately 5.44 µm to 12.56 µm, showing greater size variability than RV1. The particles appeared more densely packed with minimal apparent porosity, suggesting a more uniform composition and different aggregation properties compared to RV1 (Fig. 3b).

### Amino acid analysis and ^1^H NMR spectra

The amino acid compositions of peptide fractions RV1 and RV2 were quantified using HPLC analysis (Table 3), and their corresponding proton nuclear magnetic resonance (^1^H NMR) spectra were recorded in D_2_O to investigate the chemical environments of the constituent amino acid residues. A strong correlation was observed between the dominant amino acid residues identified by quantitative analysis and the characteristic signals present in the ^1^H NMR spectra for each peptide fraction.

**Table 3:** The comparison between RV1 and RV2 using amino acid analysis and proton nuclear magnetic resonance (^1^H NMR) spectra.

NMR chemical shift (δ, ppm)	Proton type	Associated amino acids	RV1 composition (%)	RV2 composition (%)	Interpretation
3.75–3.80	α-CH of polar and acidic residues	Serine, Glutamic acid, Alanine	Ser (11.65), Glu (23.61), Ala (19.43)	Ser (7.22), Glu (11.15), Ala (9.93)	Strongest region in both; corresponds to polar α-protons
3.55–3.65	CH_2_ of Glycine, β-CH of Glu, Pro	Glycine, Glutamic acid, Proline	Glu (23.61), Pro (2.31)	Gly (20.47), Glu (11.15), Pro (11.43)	High intensity in RV2 due to Gly and Pro richness
3.30–3.50	CH_2_ in polar side chains and α-CH	Glutamic acid, Threonine, Proline	Glu, Thr (2.76), Pro	Glu, Thr (4.58), Pro	Broadened multiplets in RV2, sharper in RV1
1.80–2.00	CH_2_ in aliphatic side chains	Valine, Leucine, Alanine	Val (7.11), Leu (5.55), Ala (19.43)	Val (4.89), Leu (7.03), Ala (9.93)	More intense in RV1, indicates stronger hydrophobic contribution
1.00–1.30	Methyl (CH_3_) protons in aliphatic AAs	Valine, Leucine, Isoleucine, Alanine	Val, Leu, Ala	Val, Leu, Ala	Aliphatic methyl peaks sharper in RV1 due to higher Ala/Val

### RV1 composition and NMR analysis

The amino acid analysis of RV1 revealed a composition rich in acidic and polar residues, particularly glutamic acid (23.61%), alanine (19.43%), and serine (11.65%), along with significant amounts of hydrophobic residues including valine (7.11%) and leucine (5.55%) (Table 3). This mixed polar/hydrophobic composition suggests potential amphipathic characteristics that may contribute to membrane-interactive properties.

The ^1^H NMR spectrum of RV1 reflected this amino acid composition, with the most intense signals observed between δ 3.75–3.80 ppm, corresponding to the α-CH protons of the abundant polar and acidic amino acids (glutamic acid, serine, alanine). Additional signals associated with polar side chains and α-protons were observed in the δ 3.30–3.50 ppm range, consistent with residues like glutamic acid and threonine (2.76%). The aliphatic region displayed characteristic signals for methyl (CH_3_) protons between δ 1.00–1.30 ppm and methylene (CH_2_) protons between δ 1.80–2.00 ppm. The intensity and sharpness of these signals, particularly the methyl peaks, were consistent with the relatively high percentages of alanine, valine, and leucine found in RV1.

### RV2 composition and NMR analysis

Peptide fraction RV2 exhibited a markedly different amino acid profile, characterized by high percentages of glycine (20.47%) and proline (11.43%), alongside significant amounts of glutamic acid (11.15%) and alanine (9.93%) (Table 3). This unique composition, particularly the high glycine and proline content, suggests distinct structural and conformational properties compared to RV1.

The ^1^H NMR spectrum of RV2 showed greater complexity reflecting this unique composition. Intense signals were observed between δ 3.55–3.65 ppm, attributable to the CH_2_ protons of the abundant glycine and potentially overlapping signals from β-CH protons of glutamic acid and proline. The presence of proline (11.43%) contributed to distinct, somewhat broadened multiplets observed in the δ 3.30–3.50 ppm region, alongside signals from glutamic acid and threonine (4.58%). Compared to RV1, the signals in the α-proton region (δ 3.75–3.80 ppm) showed more complex patterns influenced by the high glycine and proline content. The aliphatic region (δ 1.00–1.30 ppm and δ 1.80–2.00 ppm) showed signals corresponding to valine (4.89%), leucine (7.03%), and alanine (9.93%), but with comparatively lower intensity than in RV1, reflecting the reduced proportion of these hydrophobic residues in RV2.

### Cell cycle analysis

**Fig. 4:**
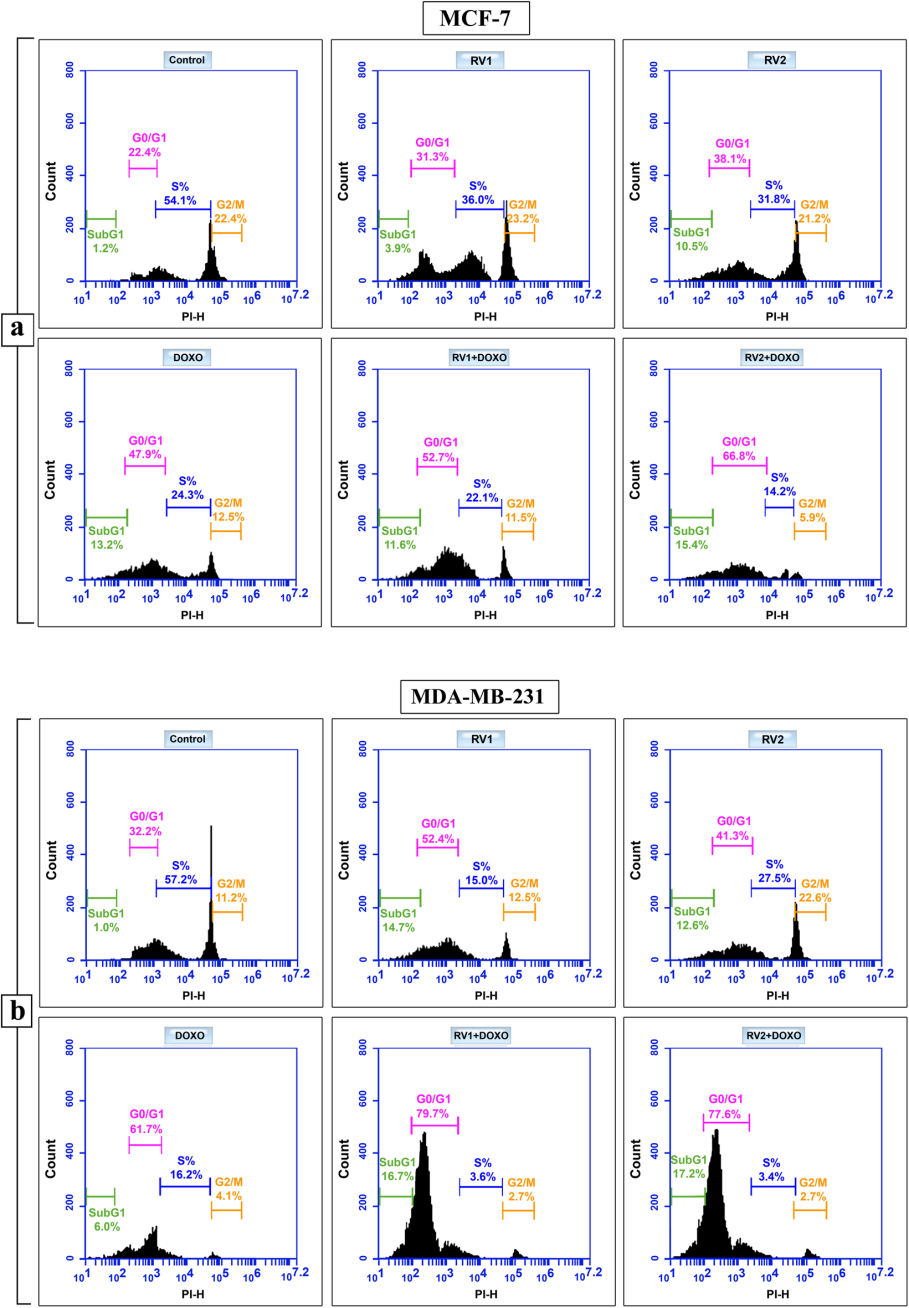
Effects of RV1, RV2, DOXO, and combinations on cell cycle distribution in human breast cancer cells: Cells were treated with IC_50_ concentrations of RV1, RV2, and DOXO (alone or in combination) for 72 h, stained with Propidium Iodide (PI), and analyzed by flow cytometry. Representative histograms display cell cycle phase distribution (SubG1, G0/G1, S, G2/M) for (**a**) MCF-7 cells and (**b**) MDA-MB-231 cells under various treatment conditions compared to untreated controls. Percentages for each phase are indicated.

The impact of peptide fractions RV1 and RV2, as well as DOXO, on cell cycle progression was investigated in MCF-7 and MDA-MB-231 breast cancer cell lines using flow cytometry analysis of propidium iodide (PI) stained cells after 72 h of treatment at IC_50_ concentrations (Fig. 4; Supplementary Table S1).

### MCF-7 cell cycle effects

Untreated control MCF-7 cells exhibited a distribution primarily within the S phase (54.1%), with smaller populations in G0/G1 (22.4%) and G2/M (22.4%) phases (Fig. 4a; Supplementary Table S1). Treatment with RV1 at its IC_50_ concentration induced notable cell cycle arrest at the G0/G1 checkpoint, evidenced by an increase in the G0/G1 population to 31.3% and a concomitant decrease in the S phase population to 36.0%. A slight increase in the G2/M phase (23.2%) and the appearance of a SubG1 population (3.9%), indicative of apoptotic cells with fragmented DNA, was also observed (Fig. 4a; Supplementary Table S1).

RV2 treatment similarly arrested MCF-7 cells in G0/G1 phase (38.1%), reducing the S phase population to 31.8% and decreasing the G2/M phase to 21.2%, accompanied by a substantial increase in the SubG1 fraction (10.5%). DOXO treatment resulted in stronger G0/G1 arrest (47.9%), with reductions in both S phase (24.3%) and G2/M phase (12.5%), and a significant SubG1 population (13.2%) (Fig. 4a; Supplementary Table S1).

### MDA-MB-231 cell cycle effects

Control MDA-MB-231 cells were predominantly in S phase (57.2%), with 32.2% in G0/G1 and 11.2% in G2/M (Fig. 4b and Supplementary Table S2). In MDA-MB-231 cells, RV1 treatment caused pronounced G0/G1 arrest, increasing this phase population to 52.4% while sharply reducing the S phase population to 15.0%. The G2/M phase remained relatively stable (12.5%), but a substantial SubG1 fraction (14.7%) emerged, indicating significant apoptosis induction (Fig. 4b; Supplementary Table S2).

RV2 treatment also induced G0/G1 arrest (41.3%), decreased S phase (27.5%), and notably increased the G2/M population (22.6%), along with a significant SubG1 fraction (12.6%). DOXO treatment led to strong G0/G1 accumulation (61.7%), reduced S phase (16.2%) and G2/M phase (4.1%), and a moderate SubG1 population (6.0%) (Fig. 4b; Supplementary Table S2).

#### Synergistic effects of combination treatments

Combination treatments involving either RV1 or RV2 with DOXO resulted in substantially enhanced G0/G1 arrest and increased SubG1 populations compared to single treatments in both cell lines, suggesting synergistic effects on cell cycle disruption and apoptosis induction. For instance, in MCF-7 cells, RV2 + DOXO treatment led to 66.8% of cells in G0/G1 and a 15.4% SubG1 population (Supplementary Table S1). In MDA-MB-231 cells, RV1 + DOXO resulted in 79.7% G0/G1 arrest and a 16.7% SubG1 population, demonstrating the enhanced efficacy of combination therapy (Supplementary Table S2).

#### Apoptosis and necrosis induction

**Fig. 5:**
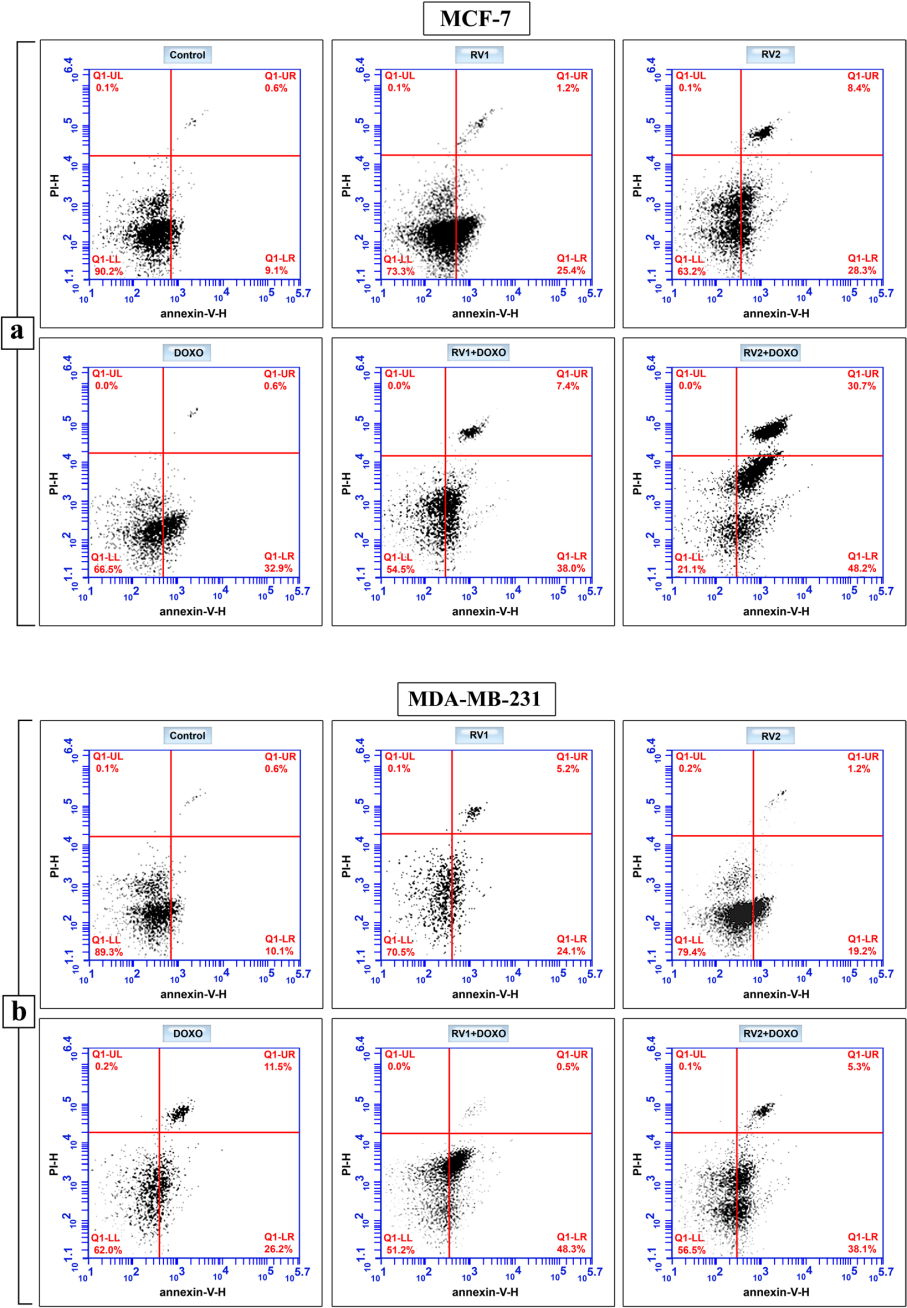
Induction of apoptosis and necrosis by RV1, RV2, DOXO, and combinations in human breast cancer cells: Cells were treated with IC_50_ concentrations of RV1, RV2, and DOXO (alone or in combination) for 72 h, stained with Annexin V-FITC and Propidium Iodide (PI), and analyzed by flow cytometry. Representative dot plots show the distribution of cells into viable (Annexin V⁻/PI⁻, lower left quadrant), early apoptotic (Annexin V⁺/PI⁻, lower right), late apoptotic/necrotic (Annexin V⁺/PI⁺, upper right), and necrotic (Annexin V⁻/PI⁺, upper left) populations for (**a**) MCF-7 cells and (**b**) MDA-MB-231 cells. Percentages for each quadrant are indicated.

To further elucidate the mechanism of cell death induced by RV1, RV2, and DOXO, apoptosis and necrosis were quantified in treated MCF-7 and MDA-MB-231 cells using Annexin V-FITC and propidium iodide (PI) dual staining followed by flow cytometry after 72 h of treatment (Fig. 5; Supplementary Tables S3, S4). Cells were categorized into four populations: viable (Annexin V⁻/PI⁻, lower left quadrant), early apoptotic (Annexin V⁺/PI⁻, lower right quadrant), late apoptotic/necrotic (Annexin V⁺/PI⁺, upper right quadrant), and necrotic (Annexin V⁻/PI⁺, upper left quadrant).

#### MCF-7 apoptosis analysis

In MCF-7 cells (Fig. 5a), the control group showed a basal level of total apoptosis (early + late apoptotic) of 9.7% (9.1% early + 0.6% late). Treatment with RV1 significantly increased the early apoptotic population to 25.4% and the late apoptotic population to 1.2%, resulting in total apoptosis of 26.6%**.** RV2 treatment induced 28.3% early apoptosis and 8.4% late apoptosis, achieving total apoptosis of 36.7%. DOXO treatment resulted in 32.9% early apoptosis and 0.6% late apoptosis with total apoptosis of 33.5% (Fig. 5a; Supplementary Table S3).

Combination treatments markedly enhanced apoptosis induction. RV1 + DOXO treatment led to 38.0% early apoptosis and 7.4% late apoptosis (total apoptosis: 45.4%), while the RV2 + DOXO combination showed the most potent effect, inducing 48.2% early apoptosis and 30.7% late apoptotic population (total apoptosis: 78.9%). The percentage of viable cells decreased from 90.2% in control to 73.1%, 63.2%, 66.5%, 54.5%, and 21.1% for RV1, RV2, DOXO, RV1 + DOXO, and RV2 + DOXO treatments, respectively. The percentage of primarily necrotic cells (Annexin V⁻/PI⁺) remained minimal (from 0.0 to 0.1% across all treatment conditions) in MCF-7 cells (Fig. 5a; Supplementary Table S3).

#### MDA-MB-231 apoptosis analysis

Similar pro-apoptotic effects were observed in MDA-MB-231 cells (Fig. 5b). The control group exhibited 10.7% total apoptosis (10.1% early + 0.6% late). RV1 treatment increased early apoptosis to 24.1% and late apoptosis to 5.2% (total apoptosis: 29.3%). RV2 treatment resulted in 19.2% early apoptosis and 1.2% late apoptosis (total apoptosis: 20.4%). DOXO treatment induced 26.2% early apoptosis and 11.5% late apoptosis (total apoptosis: 37.7%).

Combination treatments again demonstrated enhanced efficacy. RV1 + DOXO resulted in 48.3% early apoptosis and 0.5% late apoptosis (total apoptosis: 48.8%), while RV2 + DOXO induced 38.1% early apoptosis and 5.3% late apoptosis (total apoptosis: 43.4%). The percentage of viable cells decreased from 89.3% in control to 70.3%, 79.4%, 62.0%, 51.2%, and 56.5% for RV1, RV2, DOXO, RV1 + DOXO, and RV2 + DOXO treatments, respectively. Consistent with the MCF-7 results, the percentage of primarily necrotic cells remained low (ranging from 0.0 to 0.2%) under all treatment conditions in MDA-MB-231 cells, confirming that apoptosis, rather than necrosis, is the primary mode of cell death induced by these treatments (Fig. 5b; Supplementary Table S4).

#### Impact of RV1 and RV2 on gene expression levels

**Fig. 6:**
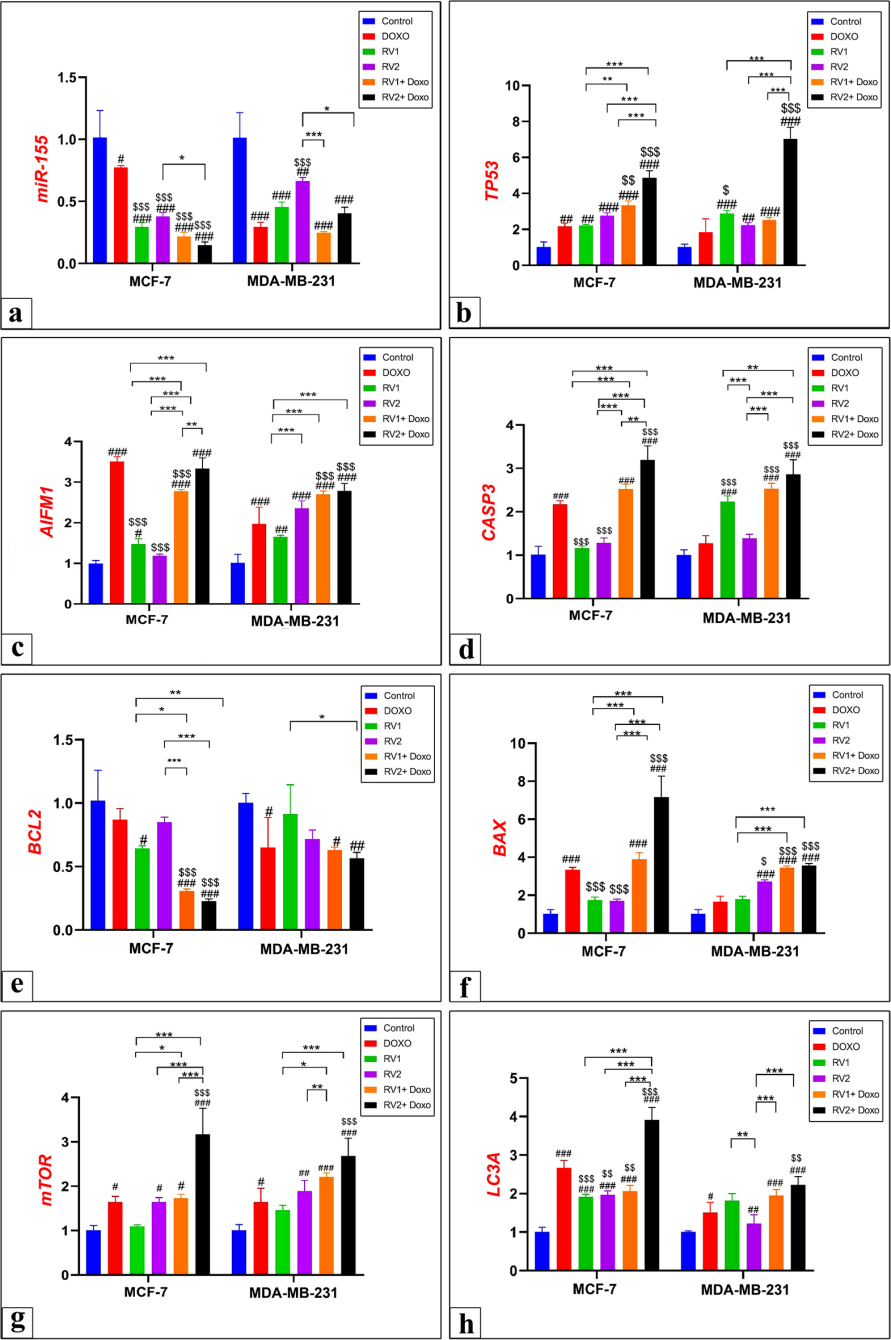
Gene expression analysis by qRT-PCR of key regulatory pathways in MCF-7 and MDA-MB-231 cells following treatment with RV1, RV2, DOXO, and their combinations. Fold changes relative to control (set as 1.0) are shown for: (**a**) *miR-155*, (**b**) *TP53*, (**c**) *AIFM1*, (**d**) *CASP3*, (**e**) *BCL2*, (**f**) *BAX*, (**g**) *mTOR*, and (**h**) *LC3A*. Data represent mean ± SE from three independent experiments. Statistical significance was determined by one-way ANOVA followed by Tukey’s post-hoc test. *p < 0.05, **p < 0.01, ***p < 0.001 versus control within each cell line; #*p* < 0.05, ##*p* < 0.01, ###*p* < 0.001 for combination treatments versus corresponding single treatments within each cell line; $*p* < 0.05, $$*p* < 0.01, $$$*p* < 0.001 for comparisons between MCF-7 and MDA-MB-231 cell lines for the same treatment.

To elucidate the molecular mechanisms underlying the cytotoxic and pro-apoptotic effects of RV1 and RV2, the relative expression levels of key genes involved in apoptosis, autophagy, and proliferation were quantified using RT-qPCR in MCF-7 and MDA-MB-231 cells following 72 h of treatment with RV1, RV2, DOXO, or combinations thereof at IC_50_ concentrations (Fig. 6; Supplementary Table S5). The results revealed significant and often cell-type-specific alterations in gene expression profiles induced by the treatments.

#### Modulation of oncogenic microRNA

Expression of the oncogenic microRNA *miR-155* was assessed to evaluate the peptides’ effects on oncogenic signaling (Fig. 6a; Supplementary Table S5). Both RV1 and RV2 significantly downregulated *miR-155* expression in MCF-7 cells, with RV1 showing more pronounced suppression than RV2. DOXO treatment also significantly reduced *miR-155* levels in MCF-7 cells. In MDA-MB-231 cells, all single treatments caused downregulation, with DOXO showing the strongest effect. Combination treatments (RV1 + DOXO and RV2 + DOXO) resulted in enhanced suppression compared to single agents in both cell lines, with RV2 + DOXO showing significantly different effects between cell lines (*p* = 0.0170).

#### Effects on pro-apoptotic pathway genes

Both RV1 and RV2 significantly upregulated *TP53* expression in both cell lines compared to controls, while DOXO treatment increased TP53 levels but without reaching statistical significance (Fig. 6b; Supplementary Table S5). Combination treatments generally resulted in higher *TP53* expression, with RV2 + DOXO showing the strongest induction and significantly different effects between cell lines (*p* < 0.0001).

*AIFM1* expression was significantly upregulated by all single treatments in MCF-7 cells, with DOXO showing the strongest effect (Fig. 6c; Supplementary Table S5). In MDA-MB-231 cells, RV1 and RV2 significantly increased *AIFM1* expression, with RV2 showing significantly higher levels than in MCF-7 cells (*p* < 0.0001). DOXO alone showed moderate upregulation that was significantly lower than in MCF-7 cells (*p* < 0.0001). Both combination treatments enhanced *AIFM1* expression in both cell lines.

*CASP3* expression showed distinct cell-type-specific responses (Fig. 6d, Supplementary Table S5). RV1 treatment significantly upregulated *CASP3* in MDA-MB-231 cells (*p* < 0.0001) but showed minimal effect in MCF-7 cells. RV2 treatment produced modest, non-significant increases in both cell lines. DOXO significantly induced *CASP3* in MCF-7 cells but not in MDA-MB-231 cells, with significant differences between cell lines (*p* < 0.0001). Both combination treatments significantly enhanced *CASP3* expression in both cell lines, with RV2 + DOXO showing significantly higher expression than RV1 + DOXO in MCF-7 cells (*p* = 0.0277).

The pro-apoptotic gene *BAX* showed differential responses between cell lines (Fig. 6f; Supplementary Table S5). In MCF-7 cells, all single treatments significantly upregulated *BAX* expression, with DOXO showing the strongest effect. Combination treatments further enhanced *BAX* levels, with RV2 + DOXO producing significantly higher expression than RV1 + DOXO (*p* < 0.0001). In MDA-MB-231 cells, only RV2 treatment significantly upregulated *BAX* expression (*p* = 0.0266), while RV1 and DOXO showed modest increases. Both combination treatments significantly enhanced *BAX* expression compared to controls and single treatments.

#### Effects on anti-apoptotic genes

The anti-apoptotic gene *BCL2* showed modest downregulation with single treatments that did not reach statistical significance in either cell line (Fig. 6e; Supplementary Table S5). However, both combination treatments resulted in significant downregulation in both cell lines, with significant differences between MCF-7 and MDA-MB-231 cells (RV1 + DOXO: *p* = 0.0489; RV2 + DOXO: *p* = 0.0361), demonstrating enhanced anti-apoptotic pathway suppression with combination therapy.

#### Effects on autophagy pathway genes

*mTOR* expression showed modest upregulation with single treatments that generally did not reach statistical significance in either cell line (Fig. 6g; Supplementary Table S5). Combination treatments resulted in enhanced *mTOR* upregulation compared to controls in both cell lines. Moreover, the autophagy marker *LC3A* was significantly upregulated by RV1 in MCF-7 cells, while RV2 treatment significantly induced *LC3A* in MCF-7 cells but showed significantly lower levels in MDA-MB-231 cells (*p* = 0.0018) (Fig. 6h; Supplementary Table S5). DOXO treatment significantly induced *LC3A* in MCF-7 cells but showed significantly lower induction in MDA-MB-231 cells (*p* < 0.0001). Both combination treatments enhanced *LC3A* expression compared to controls, with RV2 + DOXO showing the strongest induction in MCF-7 cells and significantly different effects between cell lines (*p* < 0.0001), suggesting enhanced autophagy activation with combination therapy.

## Discussion

The search for novel anticancer agents from natural sources, particularly marine organisms, continues to be a significant area of research due to the vast chemical diversity these organisms offer. Marine mollusks have evolved sophisticated biochemical defense mechanisms over millions of years, producing complex peptides and proteins that serve multiple physiological functions including antimicrobial defense and cellular protection. The exploration of these marine-derived bioactive compounds has emerged as a promising frontier in drug discovery, particularly in oncology, where the urgent need for novel therapeutic agents with improved specificity and reduced toxicity continues to drive research innovation. The invasive species *R. venosa* has emerged as a promising source of various bioactive compounds, including proteins, peptides, and polysaccharides with demonstrated therapeutic potential^29,30,41^. This study focused on isolating and characterizing peptide fractions from *R. venosa* tissue hydrolysates and evaluating their cytotoxic effects against human breast cancer cell lines.

The initial enzymatic hydrolysis of *R. venosa* tissue using pepsin, trypsin, and α-chymotrypsin proved effective in breaking down large proteins into smaller peptides, as evidenced by the disappearance of high molecular weight bands on SDS-PAGE. This approach is crucial for releasing potentially bioactive peptides that might be part of larger protein structures within the snail tissue. The original protein profile (180–10 kDa) is consistent with the diverse proteome typically found in gastropod tissues, which includes structural proteins, enzymes, and defense-related molecules^30,39,50^. Subsequent fractionation of the hydrolysate using FPLC anion exchange chromatography successfully separated the complex mixture into distinct fractions.

A particularly significant finding of our study is the demonstrated selectivity of RV1 and RV2 peptides for breast cancer cells compared to other cancer types. The comprehensive screening across four different cancer cell lines revealed that both peptides exhibited 25–95-fold higher potency against breast cancer cells (MCF-7 and MDA-MB-231) compared to colorectal (CaCo-2) and hepatocellular carcinoma (HepG2) cell lines (Table 2). This preferential activity is therapeutically significant, as it suggests potential for reduced off-target effects and improved therapeutic windows. Notably, both estrogen receptor-positive (MCF-7) and triple-negative (MDA-MB-231) breast cancer subtypes showed similar sensitivity, indicating a mechanism independent of hormone receptor status—particularly valuable given the limited treatment options for triple-negative breast cancer. The systematic screening approach employed in this study, testing all isolated fractions against multiple cancer types, provided a transparent and objective basis for selecting RV1 and RV2 for detailed mechanistic investigation, directly addressing concerns about arbitrary fraction selection.

Further cytotoxicity evaluation revealed that fractions RV1 and RV2 possess significant, dose-dependent antiproliferative effects specifically against breast cancer cells. The calculated IC_50_ values for RV1 (6.887 μg/ml for MCF-7, 7.288 μg/ml for MDA-MB-231) and RV2 (6.268 μg/ml for MCF-7, 4.886 μg/ml for MDA-MB-231) indicate moderate cytotoxic potency against the primary targets. While these potencies are lower than that of doxorubicin (IC_50_ values ~ 1.2–1.8 μg/ml), they are within the range typically observed for bioactive natural product fractions in early-stage investigations. Notably, RV2 exhibited the highest potency against the aggressive triple-negative MDA-MB-231 cell line, suggesting particular utility for this challenging breast cancer subtype.

Previous studies on *R. venosa* have primarily focused on the anticancer activities of larger molecules like hemocyanins or glycosaminoglycans^29,41^. For instance, Petrova et al.^30^ reported antitumor activity from hemolymph fractions (50–100 kDa) against various breast cancer lines, linking activity to components like peroxidase-like proteins and hemocyanin functional units. These fractions exhibited strong synergistic activity when combined with chemotherapeutic drugs cisplatin and tamoxifen^30^. Georgieva et al.^29^ demonstrated antiproliferative effects of purified hemocyanins. Therefore, the identification of cytotoxic activity specifically within lower molecular weight peptide fractions (RV1 and RV2) derived from enzymatic hydrolysis represents a potentially novel finding, suggesting that smaller peptides, distinct from the well-studied large proteins, also contribute to the overall anticancer potential of *R. venosa*.

The observation that other fractions (1A, 1B, 1C, 2A, 3B) lacked significant cytotoxicity underscores the specificity of the active components concentrated in RV1 and RV2 through the fractionation process. The differential sensitivity observed between the two cell lines suggests potential selectivity related to the distinct molecular characteristics of these cancer subtypes, warranting further investigation into the specific molecular targets or pathways affected by each peptide fraction. Marine-derived peptides from other mollusks have demonstrated comparable cytotoxic effects. Dolastatin-10, a peptide from the sea hare *Dolabella auricularia*, exhibited potent cytotoxicity against various cancer cell lines, including breast cancer^51^. Similarly, kahalalide F, a cyclic depsipeptide from the mollusk *Elysia rufescens*, showed selective cytotoxicity against hormone-independent breast cancer cells MDA-MB-231^52^.

Following the identification of peptide fractions RV1 and RV2 as possessing significant cytotoxic activity against breast cancer cell lines, further characterization was undertaken to understand their chemical nature. The morphological characterization by SEM revealed distinct microstructures for the isolated peptide fractions. RV1 typically appeared as larger, angular, block-like particles often forming clusters, whereas RV2 consisted of more heterogeneously shaped, smoother, and densely packed particles. These observed micrometer-scale structures likely represent aggregates formed during isolation or sample preparation, rather than individual peptide molecules. Such differences in morphology and aggregation patterns probably stem from underlying variations in peptide composition, size, charge, and hydrophobicity^39^. Importantly, these physical characteristics can influence peptide stability, solubility, cellular uptake mechanisms, and membrane interactions, ultimately impacting their therapeutic efficacy.

Furthermore, determining the amino acid composition and obtaining structural insights through ^1^H NMR spectroscopy are crucial steps in correlating the chemical properties of these peptide fractions with their observed biological effects. The distinct amino acid profiles and NMR spectral features observed for RV1 and RV2 provide valuable clues regarding the types of peptides enriched in each fraction and potentially explain their differential cytotoxic activities. Fraction RV1 was found to be rich in acidic and polar amino acids, notably glutamic acid (23.61%), alanine (19.43%), and serine (11.65%), while also containing considerable proportions of hydrophobic residues such as valine (7.11%) and leucine (5.55%). This mixed composition, featuring both charged/polar and hydrophobic components, suggests the potential for amphipathic characteristics within the constituent peptides, a common feature of many bioactive peptides, including anticancer peptides, which often interact with cell membranes. The ^1^H NMR spectrum of RV1 corroborated this composition, displaying strong signals in regions typical for α-protons of polar/acidic residues (δ 3.30–3.80 ppm) and significant signals in the aliphatic regions (δ 1.00–1.80 ppm) corresponding to the methyl and methylene groups of alanine, valine, and leucine.

In contrast, fraction RV2 presented a markedly different amino acid profile, dominated by glycine (20.47%) and proline (11.43%). While still containing substantial amounts of glutamic acid (11.15%) and alanine (9.93%), the high glycine and proline content is a defining feature. Glycine residues confer conformational flexibility, while proline introduces kinks and rigidity, often defining specific secondary structures like turns or polyproline helices. The ^1^H NMR spectrum of RV2 reflected this unique composition through its complexity, particularly the intense signals associated with glycine methylene protons and the distinct, broadened multiplets arising from proline residues. The presence of abundant proline is particularly noteworthy, as proline-rich antimicrobial peptides have previously been identified in the hemolymph of *R. venosa*^30^, suggesting RV2 might contain peptides related to this known class of bioactive molecules from the same organism.

These structural differences likely explain the observed differential bioactivity and physical properties. RV2 demonstrated greater cytotoxic potency, particularly against MDA-MB-231 cells, potentially due to its glycine/proline-mediated conformational adaptability facilitating target engagement. Furthermore, compositional variations may contribute to distinct aggregation states observed via SEM by influencing solubility and peptide assembly. While this work successfully correlates composition, NMR features, and bioactivity, it represents an initial characterization of complex peptide mixtures. Future studies involving advanced purification and sequencing are essential to identify the specific active sequences within RV1 and RV2 and elucidate precise structure–activity relationships.

Flow cytometry analysis revealed that both RV1 and RV2 significantly perturbed cell cycle progression in MCF-7 and MDA-MB-231 breast cancer cells, primarily inducing G0/G1 phase arrest characterized by increased G0/G1 populations and corresponding decreases in S phase populations. This G0/G1 arrest mechanism effectively halts DNA synthesis and cell proliferation, representing a common anticancer strategy employed by various marine-derived peptides. The extent of arrest varied between peptide fractions and cell lines, with RV1 inducing particularly strong G0/G1 arrest in MDA-MB-231 cells (52.4% increase), while RV2 caused both G0/G1 arrest and unique effects in this cell line. These differential responses likely reflect the interaction between the distinct chemical compositions of RV1 (glutamic acid/alanine/serine-rich) and RV2 (glycine/proline-rich) with the molecular backgrounds of different breast cancer subtypes, potentially involving modulation of key cell cycle regulators such as p53, cyclin D1, or CDK inhibitors.

Annexin V-FITC/PI dual staining confirmed that both peptide fractions induce cell death predominantly through apoptosis in both cell lines, with minimal necrosis observed. Most significantly, combination treatments of RV1 or RV2 with doxorubicin resulted in markedly enhanced apoptotic responses—with RV2 + DOXO achieving up to 78.9% total apoptosis in MCF-7 cells compared to 33.5% with doxorubicin alone. This represents a 2.4-fold enhancement in apoptotic efficacy, suggesting remarkable synergistic interactions. These synergistic effects are particularly valuable from a clinical perspective, as they may allow for dose reduction of conventional chemotherapeutics while maintaining or enhancing therapeutic efficacy—potentially decreasing systemic toxicity and improving patient quality of life. Such dose-sparing strategies are critically needed in clinical oncology, where chemotherapy-related toxicities significantly impact treatment compliance and patient outcomes. These findings are consistent with mechanisms observed for other marine-derived anticancer compounds, including cryptophycin-52, which induced cell cycle arrest and growth inhibition in human non-small cell lung carcinoma cells^53^ and demonstrated promising antitumor activity in Phase II clinical trials for ovarian cancer^54^ and lung cancer patients^55^.

The ability of RV1 and RV2 to induce cell cycle arrest and apoptosis is further supported by their effects on cellular processes, similar to marine sponge-derived hemiasterlins, which induced mitotic arrest and abnormal spindle formation via inhibition of spindle microtubule dynamics in MCF-7 cells^56^. Additionally, like sea cucumber intestine peptides, which demonstrated anti-cancer effects against MCF-7 cells by inhibiting PI3K/AKT signaling and regulating apoptosis-related genes^57^, RV1 and RV2 appear to target multiple cellular pathways to achieve their anticancer effects.

Perhaps most significantly, combination treatments of RV1 or RV2 with doxorubicin resulted in markedly enhanced G0/G1 arrest and substantially increased apoptotic populations compared to individual treatments in both cell lines. This synergistic interaction suggests complementary mechanisms of action and offers promising potential for combination therapy, potentially allowing reduced doses of conventional chemotherapeutics while maintaining or enhancing efficacy. Such synergistic effects are particularly valuable given that hemocyanin subunits and mucus from gastropod mollusks, including *R. venosa*, have previously shown anti-cancer effects against HT-29 colorectal carcinoma cells through apoptosis induction and regulation of nuclear morphology and chromatin condensation^29^, indicating the broad anticancer potential of *R. venosa*-derived bioactive compounds across different cancer types.

RT-qPCR analysis revealed that RV1 and RV2 significantly modulated the expression of apoptosis-, autophagy-, and proliferation-related genes, providing molecular validation for the observed cytotoxic effects and cell cycle perturbations. Both peptides upregulated key pro-apoptotic genes including *TP53*, *AIFM1*, *CASP3*, and *BAX* in a cell-type-specific manner, with RV1 inducing *CASP3* in MDA-MB-231 cells and RV2 upregulating *BAX* in the same cell line. These differential gene expression patterns directly correlate with the apoptosis data observed in flow cytometry analysis. *TP53*, the tumor suppressor that orchestrates cellular responses to stress by inducing cell cycle arrest, DNA repair, or apoptosis^58^, was consistently induced in both cell lines, providing the molecular basis for the observed G0/G1 cell cycle arrest and explaining how the peptides halt proliferation at this critical checkpoint.

These findings align with previous studies showing that *R. venosa* hemolymph triggered p53-dependent pathways in human breast cancer cells^30^ and are consistent with marine peptides like ILYMP from *Cyclina sinensis*, which induced apoptosis in prostate cancer cells via upregulating *BAX*/*BCL2* ratio and activating caspases 3 and 9-mediated apoptosis^59^. The upregulation of *AIFM1* by both peptides in both cell lines suggests involvement of caspase-independent apoptotic pathways. *AIFM1*, a mitochondrial flavoprotein that translocates to the nucleus upon apoptotic stimuli to induce chromatin condensation and DNA fragmentation^60^, indicates a broader impact on mitochondrial-mediated cell death mechanisms.

Similarly, the modulation of *BAX*, which promotes apoptosis by permeabilizing the mitochondrial outer membrane and facilitating cytochrome c release, alongside the downregulation of the anti-apoptotic gene *BCL2*, particularly by RV2 in MDA-MB-231 cells, shifts the cellular balance toward cell death. This pattern is consistent with studies on LKEENRRRRD from *Sepia esculenta*, which exhibited antiproliferative effects against prostate cancer cells by activating p53 and caspase-3-mediated apoptosis^61^, and supports previous findings that *R. venosa*-derived compounds increase *TP53*, *CASP3*, *AIFM1*, and *BAX* expression, promoting apoptotic induction via mitochondrial pathways^29^.

The analysis also revealed complex modulation of autophagy-related genes, with *LC3A* significantly upregulated by RV1 and RV2, as well as by doxorubicin, in MCF-7. *LC3A* serves as a marker for autophagosome formation and has been implicated in cancer cell survival under stress conditions^62^. The most pronounced *LC3A* induction occurred with combination treatments, particularly RV2 + doxorubicin, suggesting that peptide treatments trigger an autophagic response similar to conventional chemotherapy. However, the concurrent upregulation of *mTOR* by RV2 treatment and combination therapies presents a complex pattern, as *mTOR* typically functions as a negative regulator of autophagy initiation^63^.

This apparent complexity may reflect several possibilities: the upregulation of *mTOR* mRNA might not translate to increased inhibitory activity due to post-translational modifications, autophagy activation through *mTOR*-independent pathways, or accumulation of autophagosomes due to altered autophagic flux rather than increased initiation. Given that hyperactivation of *mTOR* signaling is common in cancers and supports tumor growth by inhibiting autophagy^63^, the observed *LC3A* increase might reflect a stress response or altered autophagy rather than productive, cytoprotective autophagy. The intricate interplay between apoptosis and autophagy suggests that strong apoptosis induction, particularly with combination treatments, may trigger a secondary autophagic response that could contribute to overall cytotoxicity.

Additionally, the suppression of *miR-155*, particularly in MCF-7 cells, represents another important finding. *miR-155* is frequently upregulated in cancers and acts as an oncogene by targeting tumor suppressor genes and modulating immune responses, contributing to tumor progression and metastasis^64^. Its downregulation by the peptides suggests an additional anti-oncogenic mechanism beyond direct apoptosis induction. The cell-type-specific responses observed for individual genes likely reflect the interplay between the specific peptide compositions (RV1 vs. RV2) and the distinct genetic backgrounds of MCF-7 and MDA-MB-231 cells, which also explains the differential cytotoxicity patterns observed between these cell lines. The consistent and often enhanced effects seen in combination with doxorubicin at the gene expression level provide a molecular basis for the synergistic effects observed in both cell cycle arrest and apoptosis assays, further supporting the potential of these *R. venosa* peptides in combination cancer therapy. Overall, the gene expression data strongly corroborate the phenotypic observations, demonstrating that RV1 and RV2 trigger apoptosis through multiple pathways while potentially modulating autophagy and oncogenic signaling, with apoptosis appearing to be the dominant cytotoxic mechanism.

Based on our comprehensive analysis, we propose a multi-pathway mechanism for RV1 and RV2 anticancer activity: (1) Induction of G0/G1 cell cycle arrest through *p53* pathway activation, effectively halting DNA synthesis and proliferation; (2) Activation of both intrinsic (mitochondrial) apoptotic pathways via *BAX* upregulation and *BCL2* downregulation, and extrinsic pathways through *CASP3* activation; (3) Engagement of caspase-independent apoptosis via *AIFM1* upregulation; (4) Suppression of oncogenic signaling through *miR-155* downregulation; and (5) Modulation of autophagy pathways, potentially contributing to overall cytotoxicity. The cell-type-specific variations in these responses reflect the interaction between peptide composition and the distinct molecular backgrounds of different breast cancer subtypes, providing a scientific basis for the observed differential sensitivities.

While our study provides compelling evidence for the anticancer potential of RV1 and RV2, several limitations of our current study should be acknowledged. First, the specific amino acid sequences of the active peptide fractions RV1 and RV2 remain undetermined due to resource constraints. Advanced proteomic techniques such as LC–MS/MS and MALDI-TOF would provide definitive peptide identification and are prioritized for future funded studies. Second, our UV detection at 280 nm, while adequate for our aromatic amino acid-containing fractions, may not capture the complete peptide profile compared to more sensitive 214 nm detection. Third, our characterization approach using SEM, amino acid analysis, and ^1^H NMR of peptide mixtures provides preliminary information but lacks the specificity of modern analytical techniques. Despite these limitations, the consistent bioactivity profiles and reproducible cytotoxic effects across independent experiments validate the pharmaceutical relevance of our isolated fractions. Importantly, our screening results demonstrate preferential cytotoxicity of RV1 and RV2 against breast cancer cells compared to other cancer types (30–60-fold higher potency against MCF-7 and MDA-MB-231 versus CaCO-2 and HepG2 cells), suggesting tissue-specific or receptor-mediated mechanisms that warrant further investigation.

## Conclusion

This investigation establishes marine-derived peptides RV1 and RV2 from *R. venosa* as promising anticancer agents with significant therapeutic potential for breast cancer treatment. The peptides demonstrate preferential selectivity for breast cancer cells (25–95-fold vs. other cancer types), broad-spectrum activity against both hormone receptor-positive and triple-negative subtypes, and remarkable synergistic effects with standard chemotherapy that could revolutionize combination therapy approaches. Their multi-pathway mechanism—encompassing cell cycle arrest, apoptosis induction, and oncogenic pathway suppression—positions them as valuable candidates for targeted therapy that could reduce chemotherapy doses while maintaining efficacy. The distinct amino acid compositions underlying their differential bioactivities highlight the vast therapeutic potential within marine peptide diversity. These findings not only advance our understanding of marine-derived anticancer compounds but also provide a strong foundation for clinical translation studies that could ultimately benefit breast cancer patients, particularly those with limited therapeutic options such as triple-negative breast cancer.

## Supplementary Information

Supplementary Information.

## Data Availability

Data is provided within the manuscript or supplementary information files.

## References

[1] Sung, H. et al. Global cancer statistics 2020: GLOBOCAN estimates of incidence and mortality worldwide for 36 cancers in 185 countries. *CA Cancer J. Clin.* **71**, 209–249 (2021). 33538338 10.3322/caac.21660

[2] Arnold, M. et al. Current and future burden of breast cancer: Global statistics for 2020 and 2040. *Breast* **66**, 15–23 (2022). 36084384 10.1016/j.breast.2022.08.010 PMC9465273

[3] Bray, F. et al. Global cancer statistics 2022: GLOBOCAN estimates of incidence and mortality worldwide for 36 cancers in 185 countries. *CA Cancer J. Clin.* **74**, 229–263 (2024). 38572751 10.3322/caac.21834

[4] Szymiczek, A., Lone, A. & Akbari, M. R. Molecular intrinsic versus clinical subtyping in breast cancer: A comprehensive review. *Clin. Genet.* **99**, 613–637 (2021). 33340095 10.1111/cge.13900

[5] Lammers, S. W. M. et al. The prognostic and predictive value of the luminal-like subtype in hormone receptor-positive breast cancer: An analysis of the DATA trial. *ESMO Open* **10**, 104154 (2025). 39921934 10.1016/j.esmoop.2025.104154 PMC11850755

[6] Alluri, P. & Newman, L. A. Basal-like and triple-negative breast cancers: Searching for positives among many negatives. *Surg. Oncol. Clin. N. Am.* **23**, 567–577 (2014). 24882351 10.1016/j.soc.2014.03.003 PMC4304394

[7] Waks, A. G. & Winer, E. P. Breast cancer treatment: A review. *JAMA* **321**, 288–300 (2019). 30667505 10.1001/jama.2018.19323

[8] Tommasi, C. et al. Long-term effects of breast cancer therapy and care: Calm after the storm?. *J. Clin. Med.* **11**, 7239 (2022). 36498813 10.3390/jcm11237239 PMC9738151

[9] Liu, B., Zhou, H., Tan, L., Siu, K. T. H. & Guan, X. Y. Exploring treatment options in cancer: Tumor treatment strategies. *Signal Transduct. Target. Ther.* **9**, 175 (2024). 39013849 10.1038/s41392-024-01856-7 PMC11252281

[10] Langeh, U., Kumar, V., Ahuja, P., Singh, C. & Singh, A. An update on breast cancer chemotherapy-associated toxicity and their management approaches. *Health Sci. Rev.* **9**, 100119 (2023).

[11] Anand, U. et al. Cancer chemotherapy and beyond: Current status, drug candidates, associated risks and progress in targeted therapeutics. *Genes Dis.* **10**, 1367–1401 (2024). 10.1016/j.gendis.2022.02.007 PMC10310991 37397557

[12] Wu, B., Zhang, B., Li, B., Wu, H. & Jiang, M. Cold and hot tumors: From molecular mechanisms to targeted therapy. *Signal Transduct. Target. Ther.* **9**, 274 (2024). 39420203 10.1038/s41392-024-01979-x PMC11491057

[13] Wang, X., Zhang, H. & Chen, X. Drug resistance and combating drug resistance in cancer. *Cancer Drug Resist.* **2**, 141–160 (2019). 34322663 10.20517/cdr.2019.10 PMC8315569

[14] Garg, P. et al. Emerging therapeutic strategies to overcome drug resistance in cancer cells. *Cancers* **16**, 2478 (2024). 39001539 10.3390/cancers16132478 PMC11240358

[15] Holohan, C., Van Schaeybroeck, S., Longley, D. B. & Johnston, P. G. Cancer drug resistance: An evolving paradigm. *Nat. Rev. Cancer* **13**, 714–726 (2013). 24060863 10.1038/nrc3599

[16] Chunarkar-Patil, P. et al. Anticancer drug discovery based on natural products: From computational approaches to clinical studies. *Biomedicines* **12**, 201 (2024). 38255306 10.3390/biomedicines12010201 PMC10813144

[17] Dias, D. A., Urban, S. & Roessner, U. A historical overview of natural products in drug discovery. *Metabolites* **2**, 303–336 (2012). 24957513 10.3390/metabo2020303 PMC3901206

[18] Atanasov, A. G. et al. Natural products in drug discovery: Advances and opportunities. *Nat. Rev. Drug Discov.* **20**, 200–216 (2021). 33510482 10.1038/s41573-020-00114-z PMC7841765

[19] Fu, X. Y. et al. Three rounds of stability-guided optimization and systematical evaluation of oncolytic peptide LTX-315. *J. Med. Chem.* **67**(5), 3885–3908 (2024). 38278140 10.1021/acs.jmedchem.3c02232

[20] Yin, H. et al. The hybrid oncolytic peptide NTP-385 potently inhibits adherent cancer cells by targeting the nucleus. *Acta Pharmacol. Sin.* **44**(1), 201–210 (2023). 35794372 10.1038/s41401-022-00939-x PMC9813345

[21] Yin, H. et al. Design, synthesis and anticancer evaluation of novel oncolytic peptide-chlorambucil conjugates. *Bioorg. Chem.* **138**, 106674 (2023). 37331169 10.1016/j.bioorg.2023.106674

[22] Hashem, S. et al. Targeting cancer signaling pathways by natural products: Exploring promising anti-cancer agents. *Biomed. Pharmacother.* **150**, 113054 (2022). 35658225 10.1016/j.biopha.2022.113054

[23] Chinnadurai, R. K. et al. Current research status of anti-cancer peptides: Mechanism of action, production, and clinical applications. *Biomed. Pharmacother.* **164**, 114996 (2023). 37311281 10.1016/j.biopha.2023.114996

[24] Nhàn, N. T. T., Yamada, T. & Yamada, K. H. Peptide-based agents for cancer treatment: Current applications and future directions. *Int. J. Mol. Sci.* **24**, 12931 (2023). 37629112 10.3390/ijms241612931 PMC10454368

[25] Qu, B. et al. Anticancer activities of natural antimicrobial peptides from animals. *Front. Microbiol.* **14**, 1321386 (2024). 38298540 10.3389/fmicb.2023.1321386 PMC10827920

[26] Hoskin, D. W. & Ramamoorthy, A. Studies on anticancer activities of antimicrobial peptides. *Biochim. Biophys. Acta* **1778**, 357–375 (2008). 18078805 10.1016/j.bbamem.2007.11.008 PMC2238813

[27] Bakare, O. O. et al. Biomedical relevance of novel anticancer peptides in the sensitive treatment of cancer. *Biomolecules* **11**, 1120 (2021). 34439786 10.3390/biom11081120 PMC8394746

[28] Banday, A. H., ul Azha, N., Farooq, R. & Sheikh, S. A. Exploring the potential of marine natural products in drug development: A comprehensive review. *Phytochem. Lett.* **59**, 124–135 (2024).

[29] Georgieva, A. et al. Hemocyanins from *Helix* and *Rapana* snails exhibit *in vitro* antitumor effects in human colorectal adenocarcinoma. *Biomedicines* **8**, 194 (2020). 32635655 10.3390/biomedicines8070194 PMC7400674

[30] Petrova, M. et al. Antitumor activity of bioactive compounds from *Rapana venosa* against human breast cell lines. *Pharmaceuticals* **16**, 181 (2023). 37259331 10.3390/ph16020181 PMC9959655

[31] Macedo, M. W. F. S. et al. Marine organisms as a rich source of biologically active peptides. *Front. Mar. Sci.* **8**, 667764 (2021).

[32] Barzkar, N., Sukhikh, S. & Babich, O. Study of marine microorganism metabolites: New resources for bioactive natural products. *Front. Microbiol.* **14**, 1285902 (2024). 38260902 10.3389/fmicb.2023.1285902 PMC10800913

[33] Schwartsmann, G., Da Rocha, A. B., Mattei, J. & Lopes, R. Marine-derived anticancer agents in clinical trials. *Expert Opin. Investig. Drugs* **12**, 1367–1383 (2003). 12882622 10.1517/13543784.12.8.1367

[34] Barreca, M. et al. Marine anticancer agents: An overview with a particular focus on their chemical classes. *Mar. Drugs* **18**, 619 (2020). 33291602 10.3390/md18120619 PMC7761941

[35] Habib, M. et al. In vitro anticancer and antimicrobial activities of copper-zinc superoxide dismutase purified from *Cellana rota* snail. *Egypt. J. Chem.* **66**, 1–10 (2023).

[36] Ngandjui, Y. A. T., Kereeditse, T. T., Kamika, I., Madikizela, L. M. & Msagati, T. A. M. Nutraceutical and medicinal importance of marine molluscs. *Mar. Drugs* **22**, 201 (2024). 38786591 10.3390/md22050201 PMC11123371

[37] Shen, Y., Huang, Z., Liu, G., Ke, C. & You, W. Hemolymph and transcriptome analysis to understand innate immune responses to hypoxia in Pacific abalone. *Comp. Biochem. Physiol. Part D Genomics Proteomics* **30**, 102–112 (2019). 30822665 10.1016/j.cbd.2019.02.001

[38] Habib, M. R. et al. *Thais savignyi* tissue extract: bioactivity, chemical composition, and molecular docking. *Pharm. Biol.* **60**, 1899–1914 (2022). 36200747 10.1080/13880209.2022.2123940 PMC9553184

[39] Ullah, H. et al. Sea conch (*Rapana venosa*) peptide hydrolysate regulates NF-κB pathway and restores intestinal immune homeostasis in DSS-induced colitis mice. *Food Sci. Nutr.* **12**, 10070–10086 (2024). 39723032 10.1002/fsn3.4410 PMC11666983

[40] Dolashka, P. et al. Antimicrobial proline-rich peptides from the hemolymph of marine snail *Rapana venosa*. *Peptides* **32**, 1477–1483 (2011). 21703315 10.1016/j.peptides.2011.05.001

[41] Gaspar-Pintiliescu, A. et al. Antioxidant and antiproliferative effect of a glycosaminoglycan extract from *Rapana venosa* marine snail. *PLoS ONE* **19**, e0297803 (2024). 38359063 10.1371/journal.pone.0297803 PMC10868805

[42] Crocetta, F. et al. Bottom-trawl catch composition in a highly polluted coastal area reveals multifaceted native biodiversity and complex communities of fouling organisms on litter discharge. *Mar. Environ. Res.* **155**, 104875 (2020). 31965977 10.1016/j.marenvres.2020.104875

[43] Dekker, H. & Orlin, Z. Check-list of Red sea Mollusca. *Spirula* **47**, 1–46 (2000).

[44] Vandepitte, L. et al. The World Register of Marine Species (WoRMS) through the looking glass: Insights from the data management team in light of the crystal anniversary of WoRMS. *Hydrobiologia* **852**, 1–22 (2025).

[45] Nazeer, R. A., Kumar, N. S. & Ganesh, R. J. *In vitro* and *in vivo* studies on the antioxidant activity of fish peptide isolated from the croaker (*Otolithes ruber*) muscle protein hydrolysate. *Peptides* **35**, 261–268 (2012). 22504498 10.1016/j.peptides.2012.03.028

[46] Laemmli, U. K. Cleavage of structural proteins during the assembly of the head of Bacteriophage T4. *Nature* **227**, 680–685 (1970). 5432063 10.1038/227680a0

[47] Bartolomeo, M. P. & Maisano, F. Validation of a reversed-phase HPLC method for quantitative amino acid analysis. *J. Biomol. Tech.* **17**, 131–137 (2006). 16741240 PMC2291777

[48] Jajić, I., Krstović, S., Glamočić, D., Jakšić, S. & Abramović, B. Validation of an HPLC method for the determination of amino acids in feed. *J. Serb. Chem. Soc.* **78**, 839–850 (2013).

[49] Livak, K. J. & Schmittgen, T. D. Analysis of relative gene expression data using real-time quantitative PCR and the 2(-Delta Delta C(T)) method. *Methods* **25**, 402–408 (2001). 11846609 10.1006/meth.2001.1262

[50] Kirilova, M. et al. Antibacterial action of protein fraction isolated from *Rapana venosa* hemolymph against *Escherichia coli* NBIMCC 8785. *Pharmaceuticals* **17**, 68 (2024). 38256901 10.3390/ph17010068 PMC10821198

[51] Gao, G., Wang, Y., Hua, H., Li, D. & Tang, C. Marine antitumor peptide dolastatin 10: Biological activity, structural modification and synthetic chemistry. *Mar. Drugs* **19**, 363 (2021). 34202685 10.3390/md19070363 PMC8303260

[52] Suárez, Y. et al. Kahalalide F, a new marine-derived compound, induces oncosis in human prostate and breast cancer cells. *Mol. Cancer Ther.* **2**, 863–872 (2003). 14555705

[53] Lu, K., Dempsey, J., Schultz, R. M., Shih, C. & Teicher, B. A. Cryptophycin-induced hyperphosphorylation of Bcl-2, cell cycle arrest and growth inhibition in human H460 NSCLC cells. *Cancer Chemother. Pharmacol.* **47**, 170–178 (2001). 11269744 10.1007/s002800000210

[54] D’Agostino, G. et al. A multicenter phase II study of the cryptophycin analog LY355703 in patients with platinum-resistant ovarian cancer. *Int. J. Gynecol. Cancer* **16**, 71–76 (2006). 16445613 10.1111/j.1525-1438.2006.00276.x

[55] Edelman, M. J. et al. Phase 2 study of cryptophycin 52 (LY355703) in patients previously treated with platinum-based chemotherapy for advanced non-small cell lung cancer. *Lung Cancer* **39**, 197–199 (2003). 12581573 10.1016/s0169-5002(02)00511-1

[56] Wei, W. et al. Sea cucumber intestinal peptide induces the apoptosis of MCF-7 cells by inhibiting PI3K/AKT pathway. *Front. Nutr.* **8**, 763692 (2021). 34970576 10.3389/fnut.2021.763692 PMC8713759

[57] Anderson, J., Coleman, J. E., Andersen, R. J. & Roberge, M. Cytotoxic peptides hemiasterlin, hemiasterlin A and hemiasterlin B induce mitotic arrest and abnormal spindle formation. *Cancer Chemother. Pharmacol.* **39**, 223–226 (1996). 10.1007/s002800050564 8996524

[58] Hernández Borrero, L. J. & El-Deiry, W. S. Tumor suppressor p53: biology, signaling pathways, and therapeutic targeting. *Biochim. Biophys. Acta Rev. Cancer* **1876**, 188556 (2021). 33932560 10.1016/j.bbcan.2021.188556 PMC8730328

[59] Yu, F. et al. A novel anti-proliferative pentapeptide (ILYMP) isolated from *Cyclina sinensis* protein hydrolysate induces apoptosis of DU-145 prostate cancer cells. *Mol. Med. Rep.* **18**, 771–778 (2018). 29767237 10.3892/mmr.2018.9019 PMC6059706

[60] Zong, L. & Liang, Z. Apoptosis-inducing factor: A mitochondrial protein associated with metabolic diseases—A narrative review. *Cardiovasc. Diagn. Ther.* **13**, 609–622 (2023). 37405018 10.21037/cdt-23-123 PMC10315422

[61] Huang, F., Jing, Y., Ding, G. & Yang, Z. Isolation and purification of novel peptides derived from Sepia ink: Effects on apoptosis of prostate cancer cell PC-3. *Mol. Med. Rep.* **16**, 4222–4228 (2017). 28731187 10.3892/mmr.2017.7068

[62] Chen, Y. et al. The clinical influence of autophagy-associated proteins on human lung cancer. *Dis. Markers* **2018**, 8314963 (2018). 29545906 10.1155/2018/8314963 PMC5818951

[63] Saxton, R. A. & Sabatini, D. M. mTOR signaling in growth, metabolism, and disease. *Cell* **168**, 960–976 (2017). 28283069 10.1016/j.cell.2017.02.004 PMC5394987

[64] Moutabian, H. et al. MicroRNA-155 and cancer metastasis: Regulation of invasion, migration, and epithelial-to-mesenchymal transition. *Pathol. Res. Pract.* **250**, 154789 (2023). 37741138 10.1016/j.prp.2023.154789

