# Supplementary material for: Marine-derived peptides from Rapana venosa inhibit breast cancer cell growth through synergistic mechanisms with doxorubicin
Source: Sci Rep. 2025 Sep 12;15:32451. doi: 10.1038/s41598-025-18052-4 (PMC12432159; doi:10.1038/s41598-025-18052-4)

Supplementary Table S1. Distribution of cell cycle phases in MCF-7 cells following treatment with the IC_{50} concentration of extracted peptides and doxorubicin (DOXO).

MCF-7	Control	RV1	RV2	DOXO	RV1+DOXO	RV2+DOXO
**G0/G1 Phase**	22.4 ± 0.79	31.3 ± 1.1**	38.1 ± 1.6*	47.9 ± 0.82**	52.7 ± 1.4**	66.8 ± 1.1***
**S Phase**	54.1 ± 1.2	36.0 ± 1.3*	31.8 ± 1.8*	24.3 ± 1.3***	22.1 ± 0.7**	14.2 ± 0.46***
**G2/M Phase**	22.4 ± 2.1	23.2 ± 1.2	21.2 ± 1.5	12.5 ± 0.76*	11.5 ± 0.66*	5.9 ± 0.95*

Supplementary Table S2. Distribution of cell cycle phases in MDA-MB-231 cells following treatment with the IC_{50} concentration of extracted peptides and doxorubicin (DOXO).

MDA-MB-231	Control	RV1	RV2	DOXO	RV1+DOXO	RV2+DOXO
**G0/G1 Phase**	32.2 ± 0.47	52.4 ± 1.3**	41.3 ± 0.79*	61.7± 0.46***	79.7 ± 0.40***	77.6 ± 0.46***
**S Phase**	57.2 ± 0.66	15.0 ± 0.5 [illegible]	27.5± 0.6***	16.2 ± 0.66***	3.6 ± 0.62***	3.40 ± 0.76***
**G2/M Phase**	11.2 ± 0.70	12.5 ± 0.82	22.6 ± 0.46*	4.1 ± 0.4*	2.7 ± 0.62**	2.7 ± 0.46*

Supplementary Table S3. Apoptosis and necrosis distribution in MCF-7 cells after treatment with the IC_{50} concentration of extracted peptides and doxorubicin (DOXO).

MCF-7	Control	RV1	RV2	DOXO	RV1+DOXO	RV2+DOXO
**Viable**	90.2 ± 1.67	73.3±6.1	63.2 ± 2.82*	66.5 ± 0.99*	54.5 ± 1.81***	21.1 ± 1.8***
**Early Apoptosis**	9.1 ± 0.56	25.4±0.79**	28.3 ± 1.51**	32.9 ± 1.28***	38.0 ± 1.25**	48.2 ±2.26**
**Late Apoptosis**	0.6 ± 0.141	1.2 ± 0.361	8.4 ± 0.889*	0.6 ± 0.265	7.4 ± 0.917*	30.7 ±1.51**
**Necrosis**	0.1 ± 0.026	0.1 ± 0.036	0.1 ± 0.053	0.00 ± 0.00	0.00± 0.00	0.00 ±0.00

Supplementary Table S4. Apoptosis and necrosis distribution in MDA-MB-231 cells after treatment with the IC_{50} concentration of extracted peptides and doxorubicin (DOXO).

MDA-MB-231	Control	RV1	RV2	DOXO	RV1+DOXO	RV2+DOXO
**Viable**	89.3 ± 0.92	70.5± 0.82**	79.4 ± 0.62**	62.0 ± 0.79**	51.2 ± 0.46***	56.5 ± 0.6***
**Early Apoptosis**	10.1 ± 0.3	24.1±0.56**	19.2 ± 1.0*	26.2 ± 0.95**	48.3 ± 0.76***	38.1 ± 0.96***
**Late Apoptosis**	0.60 ± 0.26	5.2 ± 0.36**	1.2 ± 0.3	11.5 ± 0.87*	0.5 ± 0.07	5.3 ± 0.45*
**Necrosis**	0.1 ± 0.04	0.1 ± 0.05	0.2 ± 0.03	0.2 ± 0.03	0.00± 0.0	0.1 ± 0.03

Supplementary Table S5. Gene expression levels of apoptotic genes, *miRNA-155*, anti-apoptotic genes, and autophagic genes in MCF-7 and MDA-MB-231 cells following treatment with the IC_{50} concentration of extracted peptides and doxorubicin (DOXO).

miR-155	**MCF-7**	**MDA-MB-231**	**P Value**	***BAX***	**MCF-7**	**MDA-MB-231**	**P Value**
**Control**	1.02 ± 0.218	1.01 ± 0.203	**ns**	**Control**	1.02 ± 0.218	1.02 ± 0.225	**ns**
**RV1**	0.295 ± 0.034	0.453 ± 0.043	**ns**	**RV1**	1.74 ± 0.157	1.8 ± 0.135	**ns**
**RV2**	0.379 ± 0.032	0.665 ± 0.028	**0.0085**	**RV2**	1.71 ± 0.09	2.71 ± 0.101	**0.0266**
**DOXO**	0.773 ± 0.016	0.294 ± 0.038	**<0.0001**	**DOXO**	3.35 ± 0.117	1.66 ± 0.289	**0.0005**
**RV1+DOXO**	0.219 ± 0.032	0.248 ± 0.009	**ns**	**RV1+DOXO**	3.91 ± 0.349	3.45 ± 0.093	**ns**
**RV2+DOXO**	0.147 ± 0.026	0.406 ± 0.047	**0.0170**	**RV2+DOXO**	7.173 ± 1.11	3.55 ± 0.109	**<0.0001**
BCL2	**MCF-7**	**MDA-MB-231**	**P Value**	***TP53***	**MCF-7**	**MDA-MB-231**	**P Value**
**Control**	1.02 ± 0.24	1.01 ± 0.074	**ns**	**Control**	1.02 ± 0.274	1.01 ± 0.171	**ns**
**RV1**	0.644 ± 0.019	0.914 ± 0. 23	**ns**	**RV1**	2.20 ± 0.069	2.87 ± 0.187	**ns**
**RV2**	0.85 ± 0.039	0.716 ± 0.071	**ns**	**RV2**	2.75 ± 0.167	2.24 ± 0.133	**ns**
**DOXO**	0.871 ± 0.086	0.651 ± 0.237	**ns**	**DOXO**	2.17 ± 0.169	1.84 ± 0.735	**ns**
**RV1+DOXO**	0.307 ± 0.016	0.629 ± 0.024	**0.0489**	**RV1+DOXO**	3.34 ± 0.254	2.52 ± 0.129	**ns**
**RV2+DOXO**	0.227 ± 0.019	0.566 ± 0.046	**0.0361**	**RV2+DOXO**	4.87 ± 0.386	7.02 ± 0.661	**<0.0001**
AIFM1	**MCF-7**	**MDA-MBA-231**	**P Value**	***CASP3***	**MCF-7**	**MDA-MB-231**	**P Value**
**Control**	1.01 ± 0.071	1.01 ± 0.208	**ns**	**Control**	1.01 ± 0.195	1.01 ± 0.119	**ns**
**RV1**	1.48 ± 0.13	1.65 ± 0.044	**ns**	**RV1**	1.17 ± 0.049	2.24 ± 0.129	**<0.0001**
**RV2**	1.19 ± 0.038	2.36 ± 0.184	**<0.0001**	**RV2**	1.28 ± 0.115	1.39 ± 0.092	**ns**
**DOXO**	3.51 ± 0.116	1.97 ± 0.413	**<0.0001**	**DOXO**	2.17 ± 0.079	1.28 ± 0.175	**<0.0001**
**RV1+DOXO**	2.78 ± 0.043	2.70 ± 0.078	**ns**	**RV1+DOXO**	2.53 ± 0.109	2.53 ± 0.128	**ns**
**RV2+DOXO**	3.34 ± 0.263	2.79 ± 0.175	**0.0088**	**RV2+DOXO**	3.19 ± 0.327	2.86 ± 0.336	**0.0277**
mTOR	**MCF-7**	**MDA-MB-231**	**P Value**	***LC3A***	**MCF-7**	**MDA-MB-231**	**P Value**
**Control**	1.01 ± 0.111	1.01 ± 0.128	**ns**	**Control**	1.01 ± 0.118	1.00 ± 0.032	**ns**
**RV1**	1.11 ± 0.037	1.46 ± 0.114	**ns**	**RV1**	1.91 ± 0.071	1.81 ± 0.191	**ns**
**RV2**	1.64 ± 0.105	1.89 ± 0.243	**ns**	**RV2**	1.97 ± 0.107	1.22 ± 0.235	**0.0018**
**DOXO**	1.65 ± 0.126	1.64 ± 0.314	**ns**	**DOXO**	2.67 ± 0.185	1.51 ± 0.258	**<0.0001**
**RV1+DOXO**	1.73 ± 0.084	2.21 ± 0.092	**ns**	**RV1+DOXO**	2.07 ± 0.144	1.96 ± 0.156	**ns**
**RV2+DOXO**	3.17 ± 0.590	2.69 ± 0.398	**ns**	**RV2+DOXO**	3.91 ± 0.327	2.23 ± 0.213	**<0.0001**

Data File C:\CHEM32\1\DATA\DR_HASSAN MASOUD 100MG_10ML 2025-01-20 18-11-32.D
Sample Name: Dr.Hassan Masoud 100mg/10ml

RV1

===

Acq. Operator : SYSTEM
Sample Operator : SYSTEM
Acq. Instrument : HPLC Location : Vial 1
Injection Date : 1/20/2025 6:15:54 PM
Inj Volume : Inj prog
Acq. Method : C:\CHEM32\1\METHODS\AMINO ACIDS_SUPELCO2.M
Last changed : 1/20/2025 10:56:56 AM by SYSTEM
(modified after loading)
Analysis Method : C:\CHEM32\1\METHODS\AMINO ACIDS_SUPELCO2.M
Last changed : 1/22/2025 1:50:39 PM by SYSTEM
(modified after loading)
Additional Info : Peak(s) manually integrated

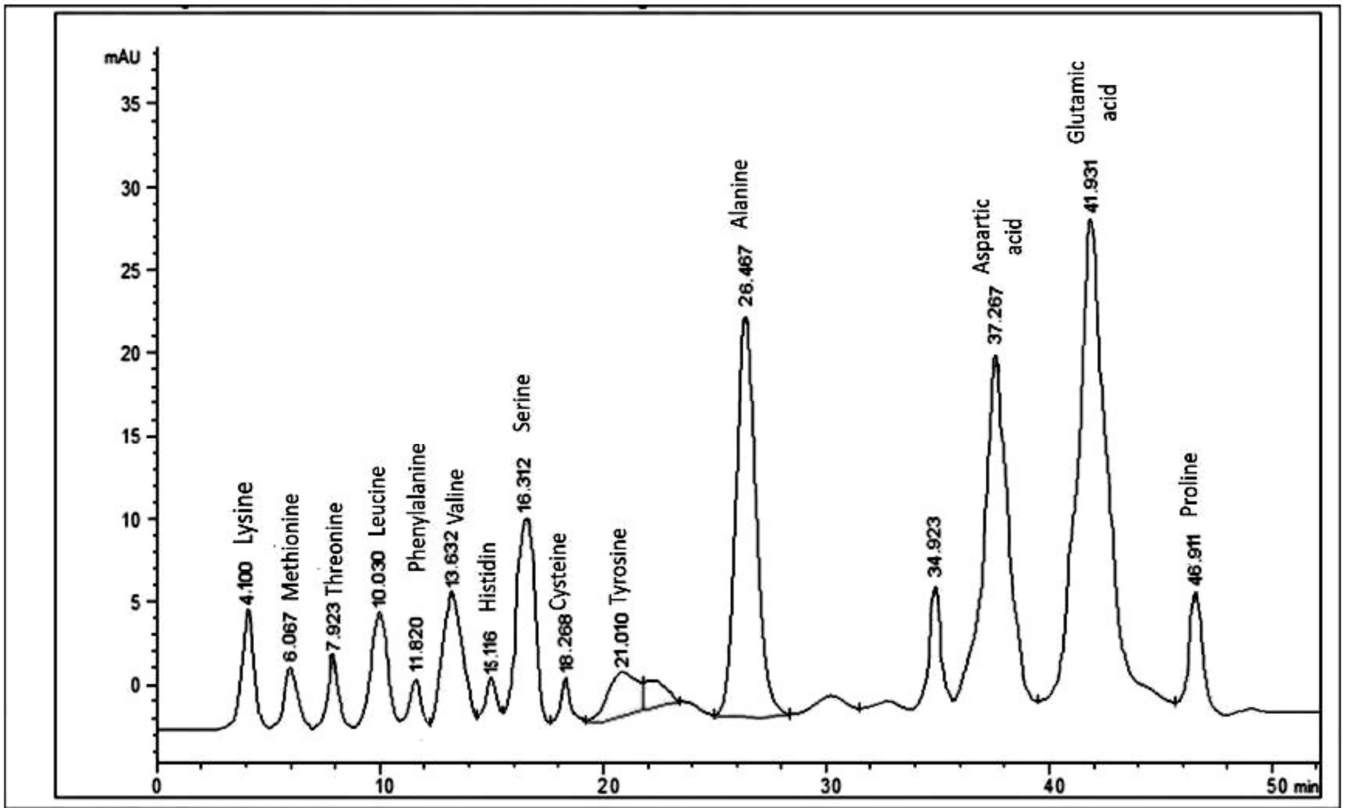

HP LaserJet 400 M401 PCL 6 on USB001
A4 210 x 297 mm/Portrait

===

Area Percent Report

===

Sorted By : Signal
Calib. Data Modified : 1/22/2025 1:50:38 PM
Multiplier : 1.0000
Dilution : 1.0000
Use Multiplier & Dilution Factor with ISTDs

Signal 1: DAD1 A, Sig=338,10 Ref=390,20

Peak #	RetTime [min]	Type	Width [min]	Area [mAU*s]	Area %	Name
1	21.090		0.0000	0.00000	0.0000	Cystine

Totals : 0.00000

Signal 2: DAD1 B, Sig=262,16 Ref=324,8

Signal 3: FLD1 A, Ex=340, Em=450, TT

Peak #	RetTime [min]	Type	Width [min]	Area [LU*s]	Area %	Name

Peak NO #	Ret Time [min]	Type	Width [min]	Area [mUA*]	Height [mUA*]	Area %
1	4.011	BV	5.231	223.205	3.761	2.122
2	6.067	BV	5.613	111.4632	2.141	1.52
3	7.923	BV	4.326	156.35	2.652	2.761
4	10.03	BV	6.022	279.263	3.881	5.55
5	11.82	BV	4.166	60.884	0.876	3.35
6	13.632	BV	7.711	291.86	4.022	7.109
7	15.116	BV	2.112	38.929	87.333	0.78
8	16.312	BV	8.678	655.705	1.031	11.65
9	18.268	BV	1.872	57.1153	1.255	0.536
10	21.01	BV	5.011	138.89	1.316	1.15
11	26.467	BV	12.43	1468.63	21.431	19.435
12	34.923	BV	6.32	205.432	4.22	2.615
13	37.267	BV	17.671	1323.782	18.431	15.5
14	41.931	BV	17.11	1842.669	25.341	23.61
15	46.911	BV	6.871	203.451	5.01	2.312
TOTAL				7057.62		

===

*** End of Report ***

Data File C:\CHEM32\1\DATA\DR_HASSAN MASOUD 100MG_10ML 2025-01-20 18-11-32.D
Sample Name: Dr.Hassan Masoud 100mg/10ml

RV2

===

Acq. Operator : SYSTEM
Sample Operator : SYSTEM
Acq. Instrument : HPLC Location : Vial 1
Injection Date : 1/20/2025 6:15:54 PM
Inj Volume : Inj prog
Acq. Method : C:\CHEM32\1\METHODS\AMINO ACIDS_SUPELCO2.M
Last changed : 1/20/2025 10:56:56 AM by SYSTEM
(modified after loading)
Analysis Method : C:\CHEM32\1\METHODS\AMINO ACIDS_SUPELCO2.M
Last changed : 1/22/2025 1:50:39 PM by SYSTEM
(modified after loading)
Additional Info : Peak(s) manually integrated

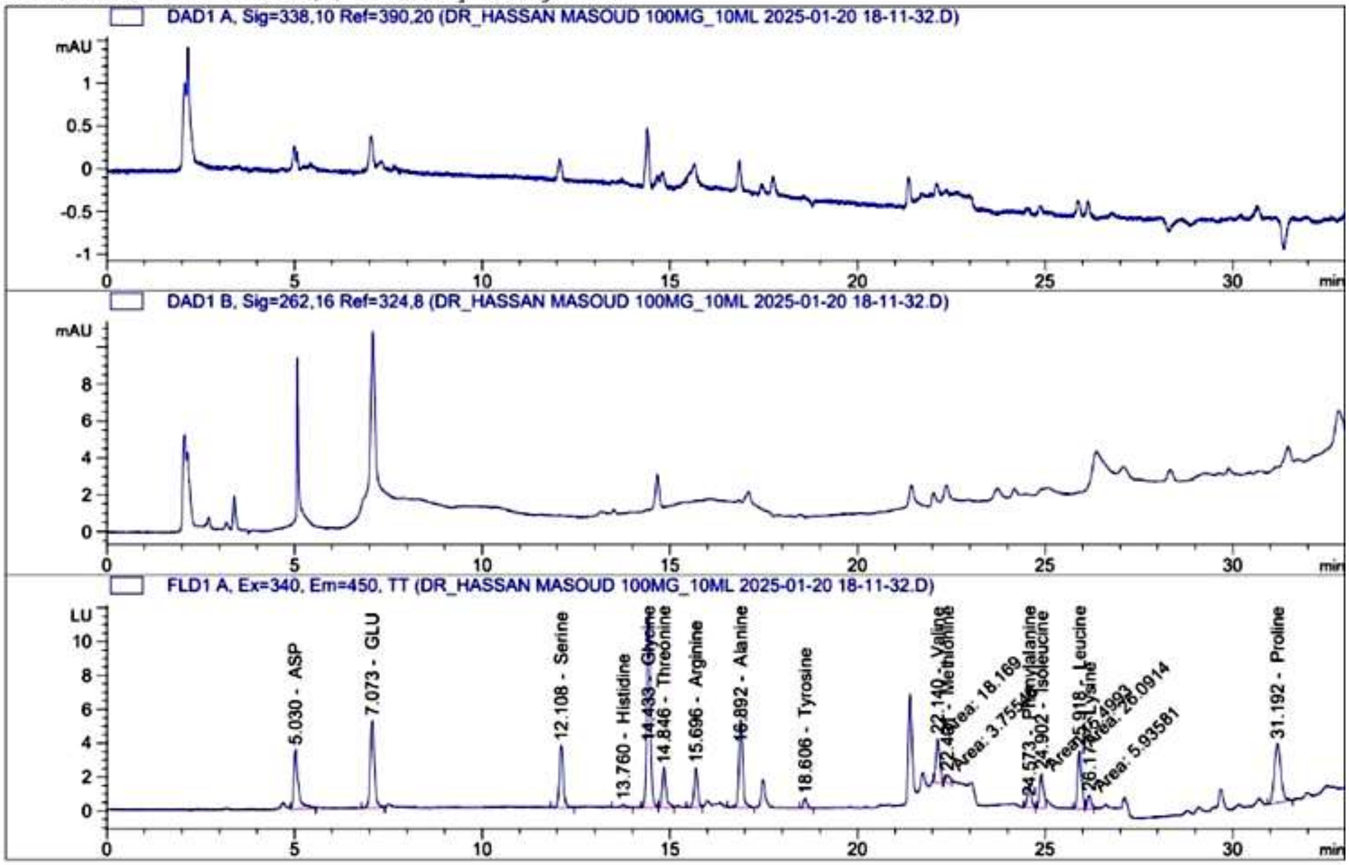

===

Area Percent Report

===

Sorted By : Signal
Calib. Data Modified : 1/22/2025 1:50:38 PM
Multiplier : 1.0000
Dilution : 1.0000
Use Multiplier & Dilution Factor with ISTDs

Data File C:\CHEM32\1\DATA\DR_HASSAN MASOUD 100MG_10ML 2025-01-20 18-11-32.D
Sample Name: Dr.Hassan Masoud 100mg/10ml

Signal 1: DAD1 A, Sig=338,10 Ref=390,20

Peak #	RetTime [min]	Type	Width [min]	Area [mAU*s]	Area %	Name
1	21.090		0.0000	0.00000	0.0000	Cystine

Totals : 0.00000

Signal 2: DAD1 B, Sig=262,16 Ref=324,8

Signal 3: FLD1 A, Ex=340, Em=450, TT

Peak #	RetTime [min]	Type	Width [min]	Area [LU*s]	Area %	Name
1	5.030	VV	0.1245	30.41483	8.1896	ASP
2	7.073	BV	0.1216	41.42319	11.1537	GLU
3	12.108	VB	0.1132	26.80269	7.2169	Serine
4	13.760	BB	0.1428	1.80158	0.4851	Histidine
5	14.433	BV	0.1064	76.02825	20.4715	Glycine
6	14.846	VB	0.1100	16.99930	4.5773	Threonine
7	15.696	BB	0.1045	15.49513	4.1723	Arginine
8	16.892	VV	0.1085	36.88089	9.9306	Alanine
9	18.606	VB	0.1098	3.99520	1.0758	Tyrosine
10	22.140	MM	0.1172	18.16898	4.8922	Valine
11	22.401	MM	0.1374	3.75548	1.0112	Methionine
12	24.573	BV	0.1173	9.63371	2.5940	Phenylalanine
13	24.902	MM	0.1281	15.49929	4.1734	Isoleucine
14	25.918	MM	0.1263	26.09142	7.0254	Leucine
15	26.177	MM	0.1236	5.93581	1.5983	Lysine
16	31.192	BV	0.1899	42.45971	11.4328	Proline

Totals : 371.38546

===

*** End of Report ***

Full length gel

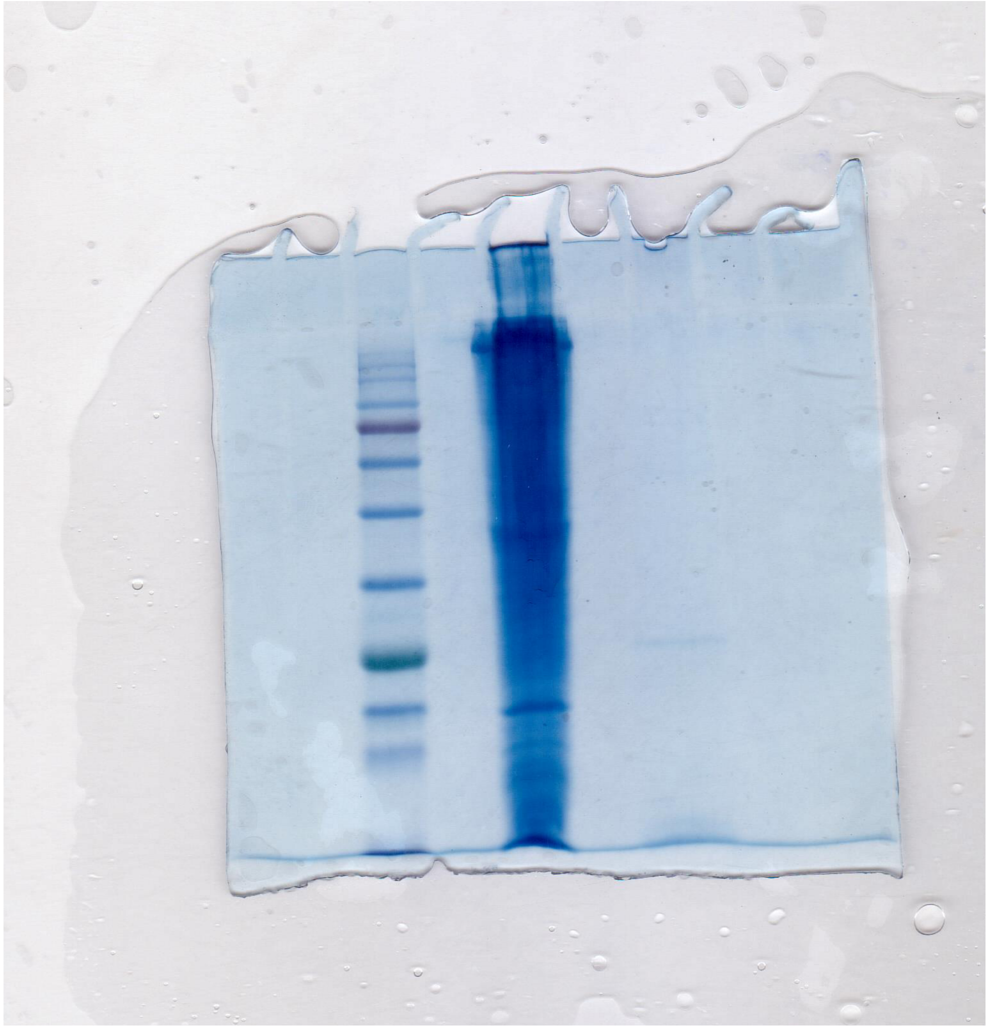

Supplement: Supplementary file 1 — Supplementary Information. [file 41598_2025_18052_MOESM1_ESM.pdf]
